# A Simple and Practical Bis-N-Heterocyclic Carbene as an Efficient Ligand in Cu-Catalyzed Glaser Reaction

**DOI:** 10.3390/molecules28135083

**Published:** 2023-06-29

**Authors:** Jie Liu, Yao Zhu, Jun Luo, Ziyi Zhu, Lin Zhao, Xiaoyan Zeng, Dongdong Li, Jun Chen, Xiaobing Lan

**Affiliations:** Hunan Provincial Key Laboratory of Xiangnan Rare-Precious Metals Compounds Research and Application, School of Chemistry and Environmental Science, Xiangnan University, Chenzhou 423000, China; 17378227105@163.com (J.L.); 17375100621@163.com (Y.Z.); 17773584550@163.com (J.L.); 15616915810@163.com (Z.Z.); 15084809428@163.com (L.Z.); 17605073856@163.com (X.Z.); dongdong_live168@163.com (D.L.); jchen4174@xnu.edu.cn (J.C.)

**Keywords:** conjugated diynes, Glaser reaction, N-heterocyclic carbene, catalysis, green chemistry

## Abstract

Conjugated diyne derivatives are important scaffolds in modern organic synthetic chemistry. Using the Glaser reaction involves the coupling of terminal alkynes which can efficiently produce conjugated diyne derivatives, while the use of a stoichiometric amount of copper salts, strong inorganic base, and excess oxidants is generally needed. Developing an environmentally friendly and effective method for the construction of symmetrical 1,3-diynes compounds by Glaser coupling is still highly desirable. In this study, we present an economical method for the production of symmetric diynes starting from various terminal acetylenes in a Glaser reaction. A simple and practical bis-N-heterocyclic carbene ligand has been introduced as efficient ligands for the Cu-catalyzed Glaser reaction. High product yields were obtained at 100 °C for a variety of substrates including aliphatic and aromatic terminal alkynes and differently substituted terminal alkynes including the highly sterically hindered substrate 2-methoxy ethynylbenzene or 2-trifluoromethyl ethynylbenzene and a series of functional groups, such as trifluoromethyl group, ester group, carboxyl group, and nitrile group. The established protocol is carried out in air under base-free condition and is operationally simple. These research work suggest that bis-N-heterocyclic carbene could also an appealing ligand for Glaser reaction and provide a reference for the preparation of symmetric 1,3-diynes in industrial filed.

## 1. Introduction

Alkynes are important structural units existing in a wide range of natural products, pharmaceuticals, and material functional molecules [1,2,3]. Alkynes also as building blocks play generally an important role in organic synthesis due to the diverse reactivity [4,5,6,7]. Particularly, conjugate 1,3-dialkynes are valuable building blocks for the synthesis of linearly π-conjugated acetylenic oligomers and polymers [8,9], supramolecular materials [10,11], and substituted heterocyclic compounds [12,13,14]. In this context, the efficient synthesis method toward this goal is always the key point of chemists’ attention [15,16,17,18,19,20]. Conventionally, the widely known method of producing synthetically important 1,3-diyne derivatives by the homocoupling of various alkynyl organometallics [21,22,23]. This reliable route, however, is not atom efficient, generating a stoichiometric amount of salt byproduct. In contrast, the homocoupling of terminal alkynes, such as Glaser-Hay coupling [24,25,26] and Cadiot-Chodkiewicz coupling [27,28,29], is widely believed to be an economic and practical method to construct 1,3-diynes.

Since the pioneering work by Glaser in 1869 [30], Glaser coupling and its modifications have become one of the most widely utilized and powerful strategies for the synthesis of diverse 1,3-dialkynes [31,32,33,34]. Numerous metal catalytic systems such as palladium [35,36], cobalt [37,38], ruthenium [39], and gold, etc., [40,41,42] have been studied on this reaction. However, most of their wide applications are limited because of their higher price or instability in air. Therefore, copper-catalyzed aerobic homocoupling of terminal alkynes remains the most popular method due to copper being an abundant and inexpensive metal. Very recently, Tiwari and co-workers [25] reported a benzotriazole ligand for the cross-coupling of terminal alkynes catalyzed by CuI in the presence of K_2_CO_3_. Lankalapalli and co-workers, using a catalytic combination of a 2-azidopyridine analogue, 4-azido-5H-pyrrolo [3,2-d]pyrimidine, and CuI, afforded homocoupled products of terminal alkynes [43]. However, it usually needs a stoichiometric amount of copper salts, excess oxidants, rigid inorganic bases, and high temperature to afford low to moderate yields for aliphatic diynes. On the other hand, Glaser coupling of terminal alkynes catalyzed using copper-containing heterogeneous catalyst has also reported in very recently [44,45]. Despite the great progress, to the best of our knowledge, few catalyst systems have been reported for the homocoupling of terminal alkynes under a neutral condition with a mild condition [46]. From both academic and industrial standpoints, it is still highly desirable to develop a more simple, effective, and environmentally friendly method for the construction of symmetrical 1,3-diyne compounds by Glaser coupling.

**Scheme 1:**
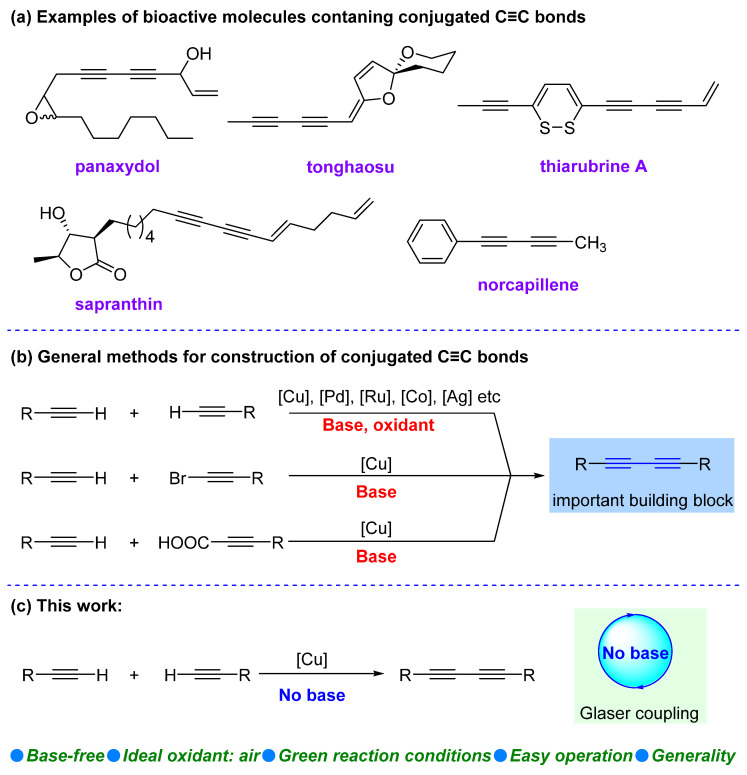
Outline of conjugate 1,3-dialkynes and they synthesis.

Using N-heterocyclic carbine (NHC) as ligands, various cross coupling reactions have been achieved under mild and environmentally friendly conditions [47,48,49,50]. So far, N-heterocyclic carbene have emerged as one of the most powerful green ligands in transition-metal catalyzed coupling reactions [51,52,53,54]. Recently, we have also demonstrated a bis-NHC as a ligand that combines a nonprecious metal that would efficiently catalyze C-C and C-N cross-coupling reactions under mild conditions [55,56]. Considering NHC ligands’ easy accessibility, environmental benignancy, and stronger σ-donors and that their steric bulkiness is tunable through variation of the N-substituents, we therefore hypothesized that we can utilize NHC as ligand for a Cu-catalyzed Glaser reaction under external-base-free conditions to produce important conjugate 1,3-diyne derivatives with broad functional group tolerance. In this context, herein, we report an efficient catalytic system with a simple and practical bis-NHC and CuCl for the Glaser coupling reaction under an air atmosphere which avoids the utilization of any base (Scheme 1). This protocol has the advantages of broad substrate scope, green reaction conditions, easy operation, and good yields.

## 2. Results and Discussion

**Scheme 2:**
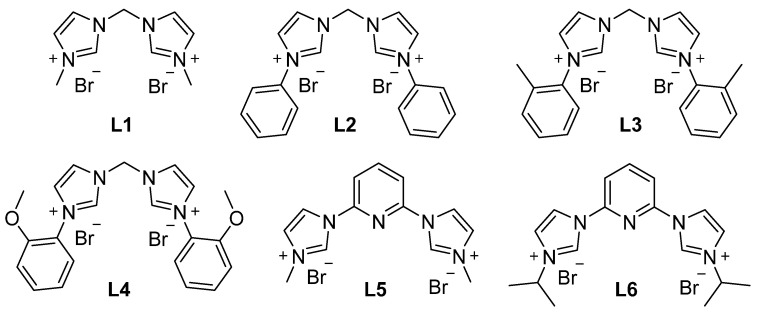
Structure of ligands.

We initially examined the reaction of phenyl acetylene **1a** under a range of reaction conditions in the presence of a CuCl catalyst (Table 1). A series of ligands were screened to evaluate their suit abilities (Scheme 2). All results are summarized in Table 1. We were pleased to find that the desired product **2a** was formed at an 86% yield when ligand **L2** was used. Other ligands, such as **L1**, **L3**, **L4**, **L5**, and **L6** were also examined and afford product **2a** with yields of 78%, 34%, 42%, 49%, and 47%, respectively (Table 1, entries 1–6). These results indicated that **L2** was most effective among the ligands **L1**–**L6**. We next studied the solvent effect because the solvent is also an important factor in this reaction. Only traces of the product were seen on the TLC plate when 1,4-dioxane was used as the solvent (Table 1, entry 7). To our delight, DMF gives the best result with a yield of 95% (Table 1, entry 8). Other solvents, such as DMAc, toluene, and xylene, also show similar reaction activities (Table 1, entry 9–11). There was no significant reduction of the yield when the temperature was 100 °C, and the reaction time could decrease to 4 h (Table 1, entry 12–16). However, further reduction of the temperature resulted in decreased product yield (Table 1, entry 17–19). Finally, control experiments indicated that both the CuCl and the ligand **L2** are essential for the reaction (Table 1, entry 20–25). Thus, it could be concluded that the optimal conditions were 5 mol% CuCl, 10 mol% **L2**, DMF as a solvent, and air as an oxidant at 100 °C for 4 h.

**Scheme 3:**
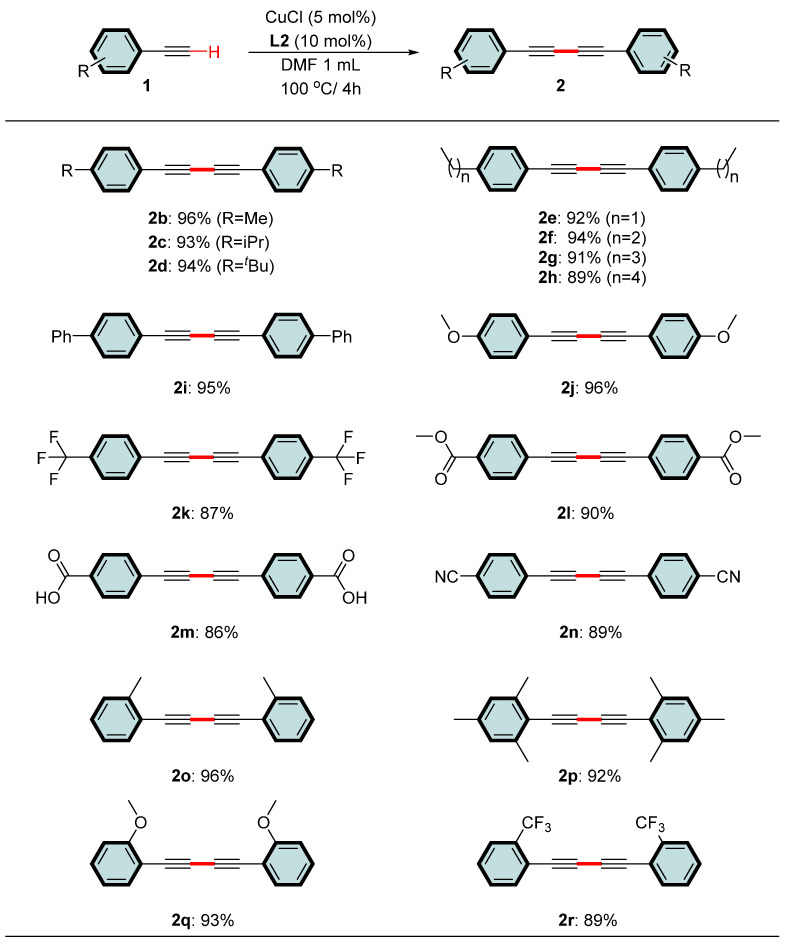
Substrate scope of aromatic terminal alkynes. Reaction conditions: **1** (1.0 mmol), Cu (5 mol%), **L2** (10 mol%), DMF (1 mL), 100 °C, under air atmosphere for 4 h. Isolated yields.

With the optimized reaction conditions in hand, the substrate scope was next explored. We first study the effect of substituents on the homocoupling of terminal alkynes. As shown in Scheme 3, various aromatic terminal alkynes with either electron-donating or electron-withdrawing substituents were well tolerated under the identified conditions, affording the desired products **2b**–**2j** with 89–96% yields. A series of functional groups, such as the trifluoromethyl group, ester group, carboxyl group, and nitrile group, were compatible with the present catalytic system. For example, 4-ethynyl-α,α,α-trifluorotoluene, methyl 4-ethynylbenzoate, 4-ethynylbenzoic acid, and 4-ethynylbenzonitrile were suitable for the couplings, and good to excellent yields of the products were obtained (86–90%). Delightedly, the sterically hindered ortho-methyl ethynylbenzene or their derivatives could provide the corresponding homocoupling product **2o** and **2p** in 96% and 92% yield, respectively. Even the highly sterically hindered substrate 2-methoxy ethynylbenzene or 2-trifluoromethyl ethynylbenzene could also react smoothly to afford the desired products **2q** and **2r** in a good yield of 93% and 89%.

**Scheme 4:**
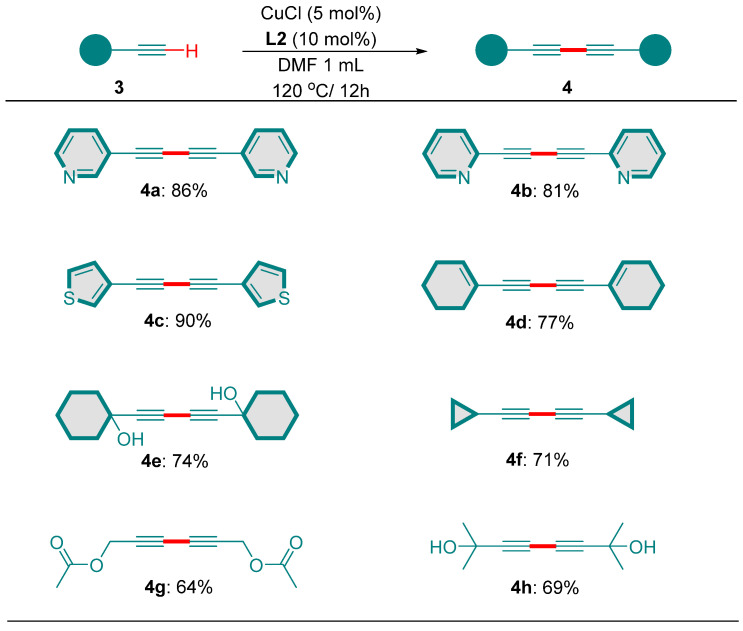
Substrate scope of aliphatic and heterocyclic terminal alkynes. Reaction conditions: **3** (1.0 mmol), Cu (5 mol%), **L2** (10 mol%), DMF (1 mL), 120 °C, under air atmosphere for 12 h. Isolated yields.

Encouraged by the above results, we explore further the scope of aliphatic terminal alkynes and heterocyclic acetylenes. It is well known that aliphatic terminal alkynes are less reactive compared to aromatic terminal alkynes. To our delight, a satisfying yield was obtained when the reaction was carried out in 120 °C with 12 h. As shown in Scheme 4, heterocyclic acetylenes, such as 3-ethynylpyridine, 2-ethynylpyridine, and 3-ethynylthiophene, can be successfully reacted to afford the corresponding compounds **4a**–**4c** in 81–90% yields. It is noteworthy that, when we employed aliphatic terminal alkynes, they performed well and provided the corresponding conjugated diyne products **4d**–**4h** in moderate yields. These results show that heterocyclic or aliphatic acetylenes are also tolerated in this present protocol.

**Scheme 5:**
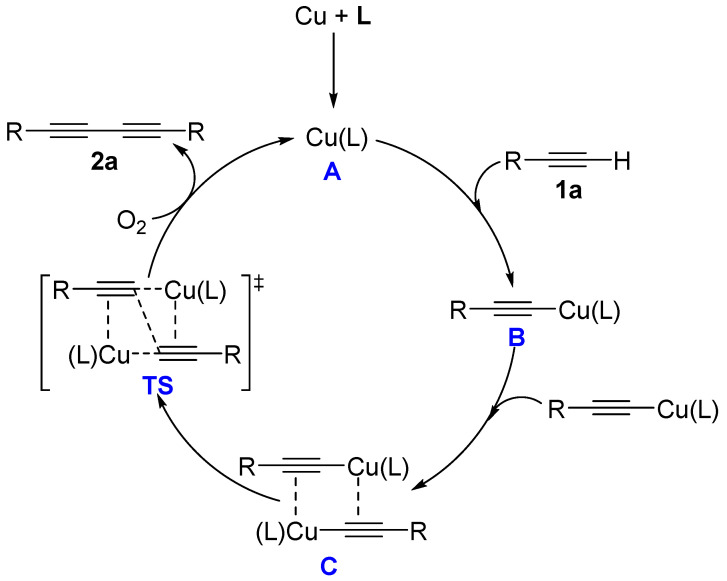
Proposed mechanism for the copper-mediated Glaser coupling.

With regard to our results, the reaction mechanism is not fully understood at present. After referring to a lot of the relevant literature [14,24,25,40,43,46], we proposed that this Cu-catalyzed Glaser homocoupling might take place as shown in Scheme 5, which possibly involves the typical Glaser reaction steps. According to our assumptions, halogen bromide anions perhaps act as Lewis bases which have a weak ability to deproton in this catalysis system. The ligand **L** was coordinated with CuCl to form complex **A**. Then, the intermediate **A** reacts with terminal alkyne to form alkynyl copper intermediate **B**, and then it involved the oxidative reaction with O_2_ to give the conjugated 1,3-diynes at the last step via intermediates **C** and transition state **TS**.

## 3. Materials and Methods

### 3.1. General Information

Unless otherwise stated, all chemicals and reagents were commercially available in analytical grade without further purification. All terminal alkynes were purchased from Aldrich Chemical Co. Ltd. (St. Louis, MI, USA). All solvents were purchased from Shanghai Macklin Chemical Co. Ltd. (Shanghai, China). **L1**–**L6** were prepared according to our previous reported procedures [49,56]. All reactions were performed under an atmosphere of air unless otherwise stated. Analytical thin layer chromatography (TLC) was performed on silica gel GF254 (layer thickness 0.20–0.25 mm). Column chromatography was carried out on silica gel (300−400 mesh) using petroleum ether as eluent. ^1^H, ^13^C NMR spectra were performed at room temperature on a Bruker Avance 400 MHz spectrometer using the residual solvent signal as internal standard (CDCl_3_: 7.26 ppm (^1^H), 77.16 ppm (^13^C); DMSO-*d*_6_: 2.50 ppm (^1^H), 39.52 ppm (^13^C)) (Supplementary Materials).

### 3.2. Typical Experimental Procedure for the Synthesis of 1,3-Diyne

Unless otherwise noted, the Glaser reaction was carried out under aerobic conditions. All solvents were used as received, and no further purification was needed. A parallel reactor containing a stir bar was charged with alkynes (1.0 mmol), CuCl (5% mol), ligands (10 mol %), and 1 mL of solvent. The reaction mixture was carried out at 100 °C for 4 h. After completion of the reaction, the reaction mixture was cooled to ambient temperature, and 10 mL of water was added. The mixture was diluted with dichloromethane (5 mL), followed by extraction three times (3 × 5 mL) with dichloromethane. The organic layer was dried with anhydrous magnesium sulfate, filtered, and evaporated under reduced pressure. The crude cross-coupling products were purified by silica-gel column chromatography using petroleum ether as eluent, and the isolated yield was then calculated. The isolated cross-coupling products were characterized by ^1^H NMR and ^13^C NMR.

### 3.3. Characterization Data of the Products

**L1** [56]. White solid. ^1^H NMR (400 MHz, DMSO-*d6*) δ 9.60 (s, 2H), 8.12 (s, 2H), 7.83 (s, 2H), 6.81 (s, 2H), 3.91 (s, 6H). ^13^C NMR (100 MHz, DMSO-*d*_6_) δ 138.03, 124.27, 121.88, 57.79, 36.28.
**L2** [57]. White solid. ^1^H NMR (400 MHz, DMSO-*d6*) δ 10.52 (s, 2H), 8.50 (d, *J* = 17.3 Hz, 4H), 7.86 (d, *J* = 7.8 Hz, 4H), 7.70 (t, *J* = 7.6 Hz, 4H), 7.63 (t, *J* = 7.3 Hz, 2H), 7.01 (s, 2H). ^13^C NMR (100 MHz, DMSO-*d*_6_) δ 137.33, 134.46, 130.28, 130.17, 123.04, 121.92, 121.50, 58.24.
**L3** [57]. White solid. ^1^H NMR (400 MHz, DMSO-*d6*) δ 10.22 (s, 2H), 8.56 (s, 2H), 8.25 (s, 2H), 7.65 (d, *J* = 7.7 Hz, 2H), 7.56 (q, *J* = 7.4 Hz, 4H), 7.49 (t, *J* = 7.2 Hz, 2H), 7.05 (s, 2H), 2.31 (s, 6H). ^13^C NMR (100 MHz, DMSO-*d*_6_) δ 138.79, 133.93, 133.20, 131.76, 130.79, 127.38, 126.31, 124.12, 122.46, 58.08, 17.30.
**L4** [57]. White solid. ^1^H NMR (400 MHz, DMSO-*d6*) δ 10.28 (s, 2H), 8.52 (s, 2H), 8.24 (s, 2H), 7.72 (dd, *J* = 7.9, 1.3 Hz, 2H), 7.66–7.57 (m, 2H), 7.41 (d, *J* = 8.3 Hz, 2H), 7.21 (t, *J* = 7.7 Hz, 2H), 7.08 (s, 2H), 3.93 (s, 6H). ^13^C NMR (100 MHz, DMSO-*d*_6_) δ 151.78, 138.77, 131.88, 125.78, 123.96, 123.03, 122.00, 121.13, 113.42, 57.97, 56.53.
**L5** [49]. White solid. ^1^H NMR (400 MHz, DMSO-*d6*) δ 10.62 (s, 2H), 8.81 (s, 2H), 8.60 (s, 1H), 8.25 (s, 2H), 8.07 (s, 2H), 4.05 (s, 6H). ^13^C NMR (100 MHz, DMSO-*d*_6_) δ 145.23, 144.89, 136.32, 125.00, 119.10, 114.04, 36.51.
**L6** [49]. White solid. ^1^H NMR (400 MHz, DMSO-*d6*) δ 10.70 (s, 2H), 8.93 (s, 2H), 8.61 (t, *J* = 8.1 Hz, 1H), 8.42–8.11 (m, 4H), 4.90 (dt, *J* = 13.3, 6.6 Hz, 2H), 1.62 (d, *J* = 6.7 Hz, 12H). ^13^C NMR (100 MHz, DMSO-*d*_6_) δ 145.31, 144.62, 134.73, 121.78, 119.66, 114.30, 53.42, 22.20.
**1,4-diphenylbuta-1,3-diyne (2a)** [24]. White solid. R_f_ = 0.6 (*n*-hexane). ^1^H NMR (400 MHz, CDCl_3_) δ 7.54 (dd, *J* = 7.8, 1.4 Hz, 4H), 7.44–7.30 (m, 6H). ^13^C NMR (100 MHz, CDCl_3_) δ 132.64, 129.35, 128.59, 121.95, 81.70, 74.06.
**1,4-di-p-tolylbuta-1,3-diyne (2b)** [24]. White solid. R_f_ = 0.6 (*n*-hexane). ^1^H NMR (400 MHz, CDCl_3_) δ 7.42 (d, *J* = 7.9 Hz, 4H), 7.14 (d, *J* = 7.8 Hz, 4H), 2.37 (s, 6H). ^13^C NMR (100 MHz, CDCl_3_) δ 139.63, 132.53, 129.35, 118.95, 81.69, 73.60, 21.76.
**1,4-bis(4-isopropylphenyl)buta-1,3-diyne (2c)** [5]. White solid. R_f_ = 0.6 (*n*-hexane). ^1^H NMR (400 MHz, CDCl_3_) δ 7.45 (d, *J* = 8.2 Hz, 4H), 7.19 (d, *J* = 8.1 Hz, 4H), 2.91 (dt, *J* = 13.8, 6.9 Hz, 2H), 1.25 (d, *J* = 6.9 Hz, 12H). ^13^C NMR (100 MHz, CDCl_3_) δ 150.46, 132.66, 126.75, 119.30, 81.70, 73.56, 34.32, 23.86.
**1,4-bis(4-(tert-butyl)phenyl)buta-1,3-diyne (2d)** [24]. White solid. R_f_ = 0.6 (*n*-hexane). ^1^H NMR (400 MHz, CDCl_3_) δ 7.45 (t, *J* = 10.7 Hz, 4H), 7.39–7.31 (m, 4H), 1.32 (s, 18H). ^13^C NMR (100 MHz, CDCl_3_) δ 152.71, 132.40, 125.61, 118.98, 81.65, 73.63, 35.06, 31.25.
**1,4-bis(4-ethylphenyl)buta-1,3-diyne (2e)** [45]. White solid. R_f_ = 0.6 (*n*-hexane). ^1^H NMR (400 MHz, CDCl_3_) δ 7.45 (d, *J* = 8.0 Hz, 4H), 7.17 (d, *J* = 8.0 Hz, 4H), 2.67 (q, *J* = 7.6 Hz, 4H), 1.24 (t, *J* = 7.6 Hz, 6H). ^13^C NMR (100 MHz, CDCl_3_) δ 145.88, 132.63, 128.16, 119.18, 81.71, 73.61, 29.06, 15.37.
**1,4-bis(4-propylphenyl)buta-1,3-diyne (2f)** [43]. White solid. R_f_ = 0.6 (*n*-hexane). ^1^H NMR (400 MHz, CDCl_3_) δ 7.44 (d, *J* = 8.1 Hz, 4H), 7.14 (d, *J* = 8.1 Hz, 4H), 2.82–2.41 (m, 4H), 1.83–1.52 (m, 4H), 0.94 (t, *J* = 7.3 Hz, 6H). ^13^C NMR (100 MHz, CDCl_3_) δ 144.37, 132.54, 128.76, 119.20, 81.73, 73.64, 38.19, 24.40, 13.89.
**1,4-bis(4-butylphenyl)buta-1,3-diyne (2g)** [25]. White solid. R_f_ = 0.6 (*n*-hexane). ^1^H NMR (400 MHz, CDCl_3_) δ 7.44 (d, *J* = 8.1 Hz, 4H), 7.15 (d, *J* = 8.2 Hz, 4H), 2.78–2.47 (m, 4H), 1.60 (dt, *J* = 12.9, 7.5 Hz, 4H), 1.35 (dq, *J* = 14.6, 7.3 Hz, 4H), 0.93 (t, *J* = 7.3 Hz, 6H). ^13^C NMR (100 MHz, CDCl_3_) δ 144.60, 132.55, 128.70, 119.15, 81.73, 73.63, 35.83, 33.45, 22.45, 14.05.
**1,4-bis(4-pentylphenyl)buta-1,3-diyne (2h)** [24]. White solid. R_f_ = 0.6 (*n*-hexane). ^1^H NMR (400 MHz, CDCl_3_) δ 7.44 (d, *J* = 8.0 Hz, 4H), 7.15 (d, *J* = 8.0 Hz, 4H), 2.98–2.39 (m, 4H), 1.78–1.52 (m, 4H), 1.32 (dt, *J* = 10.6, 3.3 Hz, 8H), 0.90 (t, *J* = 6.8 Hz, 6H). ^13^C NMR (100 MHz, CDCl_3_) δ 144.63, 132.55, 128.69, 119.14, 81.73, 73.64, 36.11, 31.58, 30.99, 22.65, 14.14.
**1,4-di([1,1′-biphenyl]-4-yl)buta-1,3-diyne (2i)** [5]. White solid. R_f_ = 0.6 (*n*-hexane). ^1^H NMR (400 MHz, CDCl_3_) δ 7.64–7.53 (m, 12H), 7.45 (t, *J* = 7.5 Hz, 4H), 7.37 (t, *J* = 7.3 Hz, 2H). ^13^C NMR (100 MHz, CDCl_3_) δ 141.74, 140.41, 132.70, 129.02, 127.87, 127.20, 127.15, 121.13, 83.70, 77.88.
**1,4-bis(4-methoxyphenyl)buta-1,3-diyne (2j)** [24]. White solid. R_f_ = 0.6 (*n*-hexane). ^1^H NMR (400 MHz, CDCl_3_) δ 7.46 (d, *J* = 8.9 Hz, 4H), 6.85 (d, *J* = 8.8 Hz, 4H), 3.82 (s, 6H). ^13^C NMR (100 MHz, CDCl_3_) δ 160.39, 134.18, 114.28, 114.11, 81.38, 73.10, 55.48.
**1,4-bis(4-(trifluoromethyl)phenyl)buta-1,3-diyne (2k)** [5]. White solid. R_f_ = 0.6 (*n*-hexane). ^1^H NMR (400 MHz, CDCl_3_) δ 7.63 (q, *J* = 8.5 Hz, 8H). ^13^C NMR (100 MHz, CDCl_3_) δ 132.96, 131.27 (d, *J* = 33.0 Hz), 125.61 (d, *J* = 3.7 Hz), 125.31 (d, *J* = 22.8 Hz), 122.49, 81.12, 75.79.
**dimethyl 4,4′-(buta-1,3-diyne-1,4-diyl)dibenzoate (2l)** [24]. White solid. Rf = 0.7 (10% ethyl acetate/*n*-hexane) ^1^H NMR (400 MHz, CDCl_3_) δ 8.02 (d, *J* = 8.4 Hz, 4H), 7.59 (d, *J* = 8.4 Hz, 4H), 3.93 (s, 6H). ^13^C NMR (100 MHz, CDCl_3_) δ 166.41, 132.62, 130.72, 129.73, 126.28, 82.00, 76.42, 52.50.
**4,4′-(buta-1,3-diyne-1,4-diyl)dibenzoic acid (2m)** [58]. White solid. R_f_ = 0.6 (ethyl acetate). ^1^H NMR (400 MHz, CDCl_3_) δ 8.06 (d, *J* = 8.2 Hz, 4H), 7.58 (d, *J* = 8.2 Hz, 4H), 3.26 (s, 2H). ^13^C NMR (100 MHz, CDCl_3_) δ 170.03, 132.33, 130.19, 80.63.
**4,4′-(buta-1,3-diyne-1,4-diyl)dibenzonitrile (2n)** [43]. White solid. R_f_ = 0.4 (*n*-hexane). ^1^H NMR (400 MHz, CDCl_3_) δ 7.74–7.52 (m, 8H). ^13^C NMR (100 MHz, CDCl_3_) δ 132.82, 132.17, 127.16, 118.39, 112.50, 82.01, 81.67.
**1,4-di-o-tolylbuta-1,3-diyne (2o)** [36]. White solid. R_f_ = 0.6 (*n*-hexane). ^1^H NMR (400 MHz, CDCl_3_) δ 7.51 (d, *J* = 7.6 Hz, 2H), 7.25 (dt, *J* = 17.1, 7.3 Hz, 4H), 7.16 (t, *J* = 7.4 Hz, 2H), 2.51 (s, 6H). ^13^C NMR (100 MHz, CDCl_3_) δ 141.78, 133.06, 129.71, 129.24, 125.80, 121.88, 81.29, 77.67, 20.88.
**1,4-dimesitylbuta-1,3-diyne (2p)** [59]. White solid. R_f_ = 0.6 (*n*-hexane). ^1^H NMR (400 MHz, CDCl_3_) δ 6.87 (s, 4H), 2.41 (s, 12H), 2.28 (s, 6H). ^13^C NMR (100 MHz, CDCl_3_) δ 140.96, 138.25, 127.71, 119.09, 84.64, 81.52, 21.45, 21.02.
**1,4-bis(2-methoxyphenyl)buta-1,3-diyne (2q)** [36]. White solid. R_f_ = 0.6 (*n*-hexane). ^1^H NMR (400 MHz, CDCl_3_) δ 7.48 (d, *J* = 7.6 Hz, 2H), 7.32 (t, *J* = 7.9 Hz, 2H), 6.90 (dd, *J* = 15.5, 8.0 Hz, 4H), 3.89 (s, 6H). ^13^C NMR (100 MHz, Chloroform-*d*) δ 161.45, 134.49, 130.65, 120.61, 111.40, 110.80, 78.78, 78.10, 55.93.
**1,4-bis(2-(trifluoromethyl)phenyl)buta-1,3-diyne (2r)** [24]. White solid. White solid. R_f_ = 0.6 (*n*-hexane). ^1^H NMR (400 MHz, CDCl_3_) δ 7.70 (t, *J* = 8.3 Hz, 4H), 7.50 (dt, *J* = 22.3, 7.5 Hz, 4H). ^13^C NMR (100 MHz, CDCl_3_) δ 135.29, 131.63, 129.27, 126.20 (q, *J* = 4.9 Hz), 124.75, 122.04, 119.90 (d, *J* = 2.8 Hz), 78.85, 78.75.
**1,4-di(pyridin-3-yl)buta-1,3-diyne (4a)** [24]. White solid. White solid. R_f_ = 0.4 (*n*-hexane). ^1^H NMR (400 MHz, CDCl_3_) δ 8.77 (s, 2H), 8.60 (d, *J* = 3.9 Hz, 2H), 7.93–7.70 (m, 2H), 7.30 (dd, *J* = 7.6, 5.0 Hz, 2H). ^13^C NMR (100 MHz, CDCl_3_) δ 153.12, 149.48, 139.69, 123.31, 119.07, 79.27.
**1,4-di(pyridin-2-yl)buta-1,3-diyne (4b)** [20]. White solid. White solid. R_f_ = 0.4 (*n*-hexane). ^1^H NMR (400 MHz, CDCl_3_) δ 8.62 (d, *J* = 4.6 Hz, 2H), 7.69 (td, *J* = 7.7, 1.4 Hz, 2H), 7.54 (d, *J* = 7.8 Hz, 2H), 7.29 (dd, *J* = 7.0, 5.5 Hz, 2H). ^13^C NMR (100 MHz, CDCl_3_) δ 150.53, 142.06, 136.35, 128.56, 123.93, 81.04, 73.35.
**1,4-di(thiophen-3-yl)buta-1,3-diyne (4c)** [24]. White solid. White solid. R_f_ = 0.4 (*n*-hexane). ^1^H NMR (400 MHz, CDCl_3_) δ 7.59 (s, 2H), 7.37–7.24 (m, 2H), 7.21–7.03 (m, 2H). ^13^C NMR (100 MHz, CDCl_3_) δ 131.37, 130.31, 125.74, 121.04, 76.70, 73.66.
**1,4-di(cyclohex-1-en-1-yl)buta-1,3-diyne (4d)** [25]. White solid. White solid. R_f_ = 0.5 (*n*-hexane). ^1^H NMR (400 MHz, CDCl_3_) δ 6.53–6.06 (m, 2H), 2.35–1.95 (m, 8H), 1.66–1.53 (m, 8H). ^13^C NMR (100 MHz, CDCl_3_) δ 138.23, 120.10, 82.84, 60.55, 28.83, 26.01, 22.27, 21.45.
**1,1′-(buta-1,3-diyne-1,4-diyl)bis(cyclohexan-1-ol) (4e)** [24]. White solid. R_f_ = 0.4 (ethyl acetate) ^1^H NMR (400 MHz, CDCl_3_) δ 2.14 (s, 2H), 1.88 (dd, *J* = 9.9, 5.8 Hz, 4H), 1.67 (dq, *J* = 11.4, 5.4, 4.9 Hz, 4H), 1.61–1.45 (m, 10H), 1.32–1.11 (m, 2H). ^13^C NMR (100 MHz, CDCl_3_) δ 87.89, 72.18, 68.61, 39.86, 25.20, 23.21.
**1,4-dicyclopropylbuta-1,3-diyne (4f) [20]**. Colorless oil. White solid. R_f_ = 0.5 (*n*-hexane). ^1^H NMR (400 MHz, CDCl_3_) δ 1.24 (ddp, *J* = 11.4, 5.4, 3.0, 2.5 Hz, 2H), 0.90–0.64 (m, 8H). ^13^C NMR (100 MHz, CDCl_3_) δ 87.87, 63.51, 8.26, −0.67.
**hexa-2,4-diyne-1,6-diyl diacetate (4g)** [20]. Colorless oil. R_f_ = 0.6 (ethyl acetate). ^1^H NMR (400 MHz, CDCl_3_) δ 4.63 (s, 4H), 2.06 (s, 6H). ^13^C NMR (100 MHz, CDCl_3_) δ 170.14, 77.74, 74.88, 51.97, 20.68.
**2,7-dimethylocta-3,5-diyne-2,7-diol (4h)** [20]. Colorless oil. R_f_ = 0.4 (ethyl acetate) ^1^H NMR (400 MHz, CDCl_3_) δ 2.13 (s, 2H), 1.49 (s, 12H). ^13^C NMR (100 MHz, CDCl_3_) δ 88.91, 70.23, 65.05, 31.35.

## 4. Conclusions

In summary, using a simple and practical bis-N-heterocyclic carbene as ligand, an efficient and green method for the Cu-catalyzed Glaser reaction to produce 1,3-conjugated dialkynes was developed. This method is carried out in simple procedure and without base additives in air conditions. Various terminal alkynes containing functional groups, such as the ester group, carboxyl group, and nitrile group, etc., could be effectively coupled to produce the corresponding conjugated 1,3-diynes with good yields. Aliphatic alkynes were also compatible in our protocol. This easily accessible system implies that N-heterocyclic carbene can serve as an alternative ligand in the Glaser reaction and offers a reference for the preparation of symmetric 1,3-diynes. This work also demonstrates the great potential of bis-N-heterocyclic carbene in the green ligand for the transition metal-catalyzed coupling reactions.

## Figures and Tables

**Table 1:** Optimization of Reaction Conditions ^[a]^.

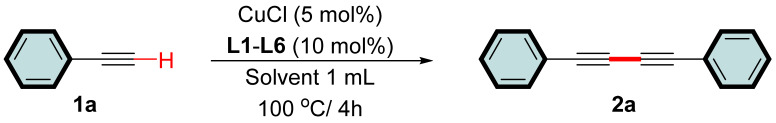						
Entry	[Cu]	Ligand	Solvent	Temperature (°C)	Time (h)	Yield(%) ^[b]^
1	CuCl	**L1**	DME	120	12	78
2	CuCl	**L2**	DME	120	12	86
3	CuCl	**L3**	DME	120	12	34
4	CuCl	**L4**	DME	120	12	42
5	CuCl	**L5**	DME	120	12	49
6	CuCl	**L6**	DME	120	12	47
7	CuCl	**L2**	1,4-dioxane	120	12	trace
8	CuCl	**L2**	DMF	120	12	95
9	CuCl	**L2**	DMAc	120	12	93
10	CuCl	**L2**	toluene	120	12	92
11	CuCl	**L2**	xylene	120	12	88
12	CuCl	**L2**	DMF	100	12	94
13	CuCl	**L2**	DMF	80	12	89
14	CuCl	**L2**	DMF	100	8	95
15	CuCl	**L2**	DMF	100	6	93
16	CuCl	**L2**	DMF	100	4	94
17	CuCl	**L2**	DMF	80	4	86
18	CuCl	**L2**	DMF	60	4	66
19	CuCl	**L2**	DMF	40	4	37
20	CuCl	-----	DMF	100	4	61
21	-----	**L2**	DMF	100	4	0
22	-----	-----	DMF	100	4	0
23	CuCl	**L2**	DMF	60	4	67
24	CuCl	-----	DMF	60	4	28
25	-----	**L2**	DMF	60	4	0

^[a]^ Reaction conditions: **1a** (1.0 mmol), Cu (5 mol %), ligand (10 mol %), Solvent (1 mL), 100 °C, under air atmosphere for 4 h. ^[b]^ Isolated yields.

## Data Availability

Not applicable.

## Supplementary Materials

The following supporting information can be downloaded at: https://www.mdpi.com/article/10.3390/molecules28135083/s1, ^1^H and ^13^C NMR spectra of the compounds.

## Sample Availability

Samples of the compounds are available from the authors.
