# Supplementary material for: A Simple and Practical Bis-N-Heterocyclic Carbene as an Efficient Ligand in Cu-Catalyzed Glaser Reaction
Source: Molecules. 2023 Jun 29;28(13):5083. doi: 10.3390/molecules28135083 (PMC10343847; doi:10.3390/molecules28135083)

Supporting Information

A Simple and Practical Bis-N-Heterocyclic Carbene as an Efficient Ligand in Cu-Catalyzed Glaser Reaction

Jie Liu, Yao Zhu, Jun Luo, Ziyi Zhu, Lin Zhao, Xiaoyan Zeng, Dongdong Li, Jun Chen and Xiaobing Lan*

Hunan Provincial Key Laboratory of Xiangnan Rare-Precious Metals Compounds Research and Application, School of Chemistry and Environmental Science, Xiangnan University, Chenzhou, 423000, PR China.

E-mail: xblan@xnu.edu.cn

Contents of supporting information

1 Copies of NMR Spectra……………………………………………………… S1

1. Copies of NMR Spectra

^{1}H NMR (400 MHz, DMSO-*d6*) and ^{13}C NMR (100 MHz, DMSO-*d6*) spectra of product L1

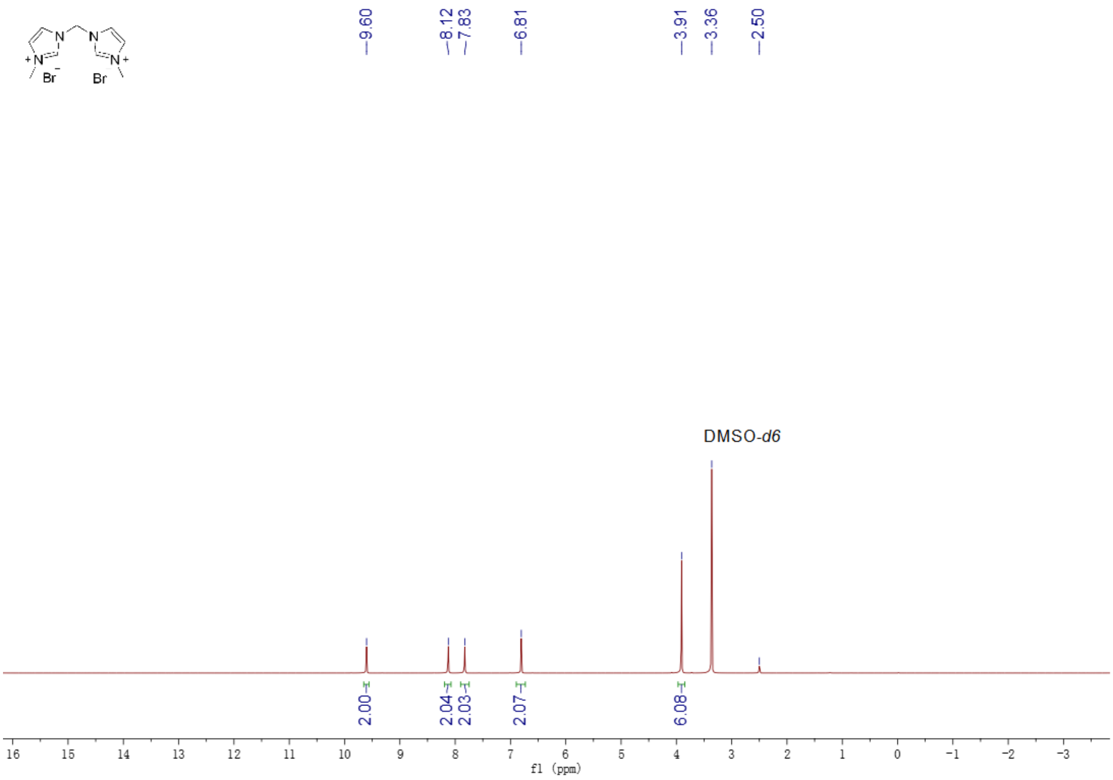

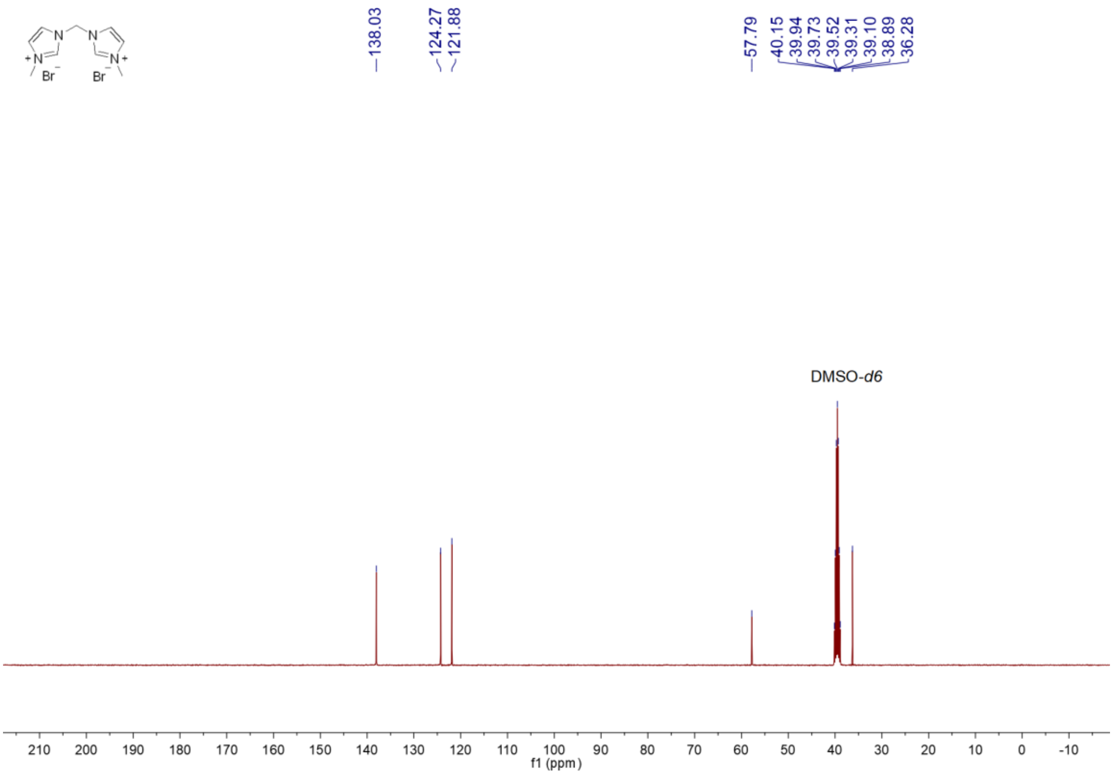

^{1}H NMR (400 MHz, DMSO-*d6*) and ^{13}C NMR (100 MHz, DMSO-*d6*) spectra of product L2

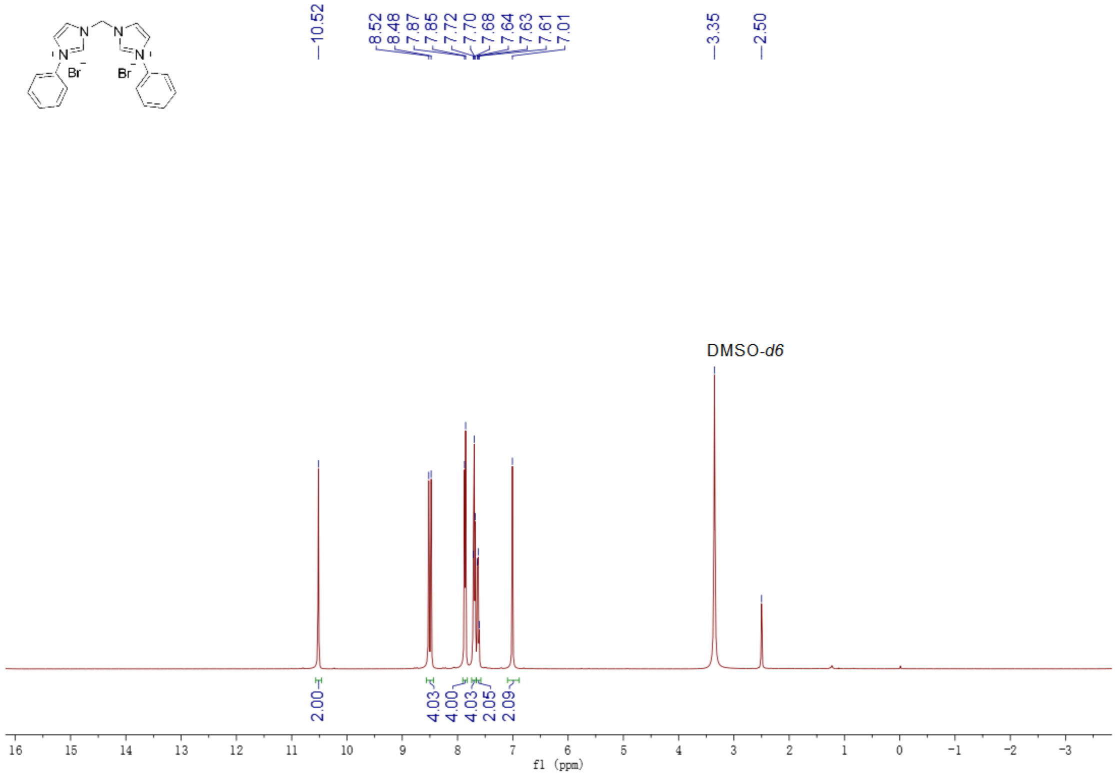

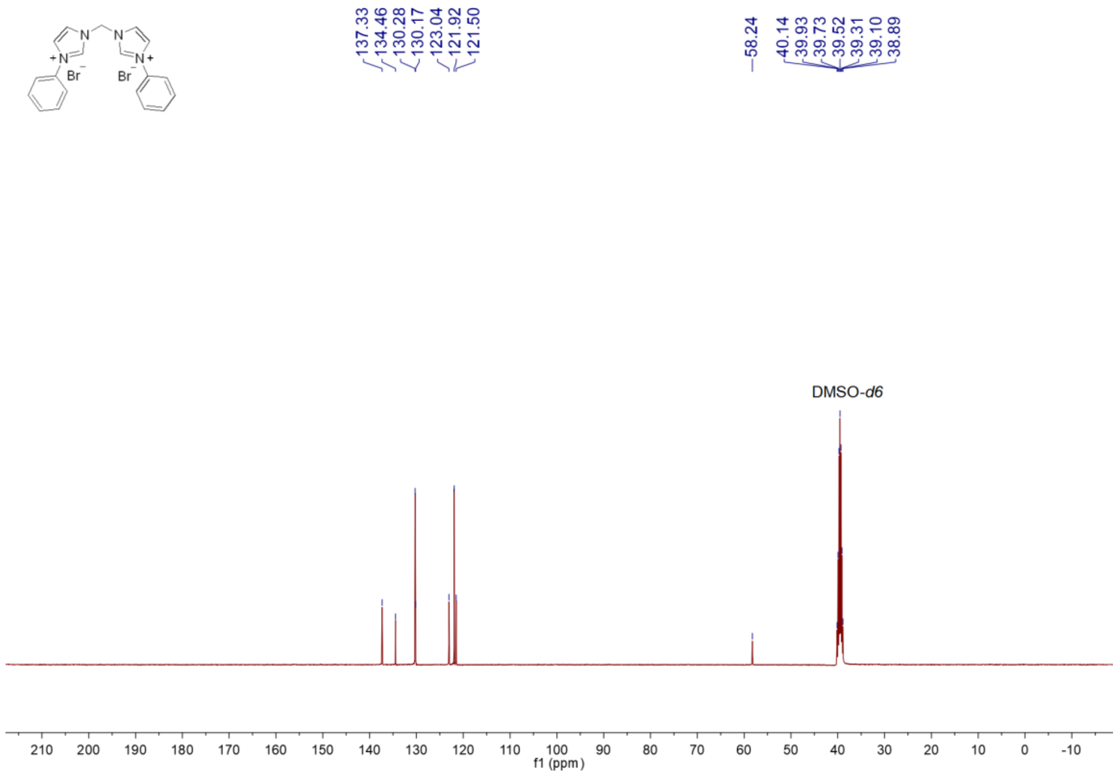

^{1}H NMR (400 MHz, DMSO-*d6*) and ^{13}C NMR (100 MHz, DMSO-*d6*) spectra of product L3

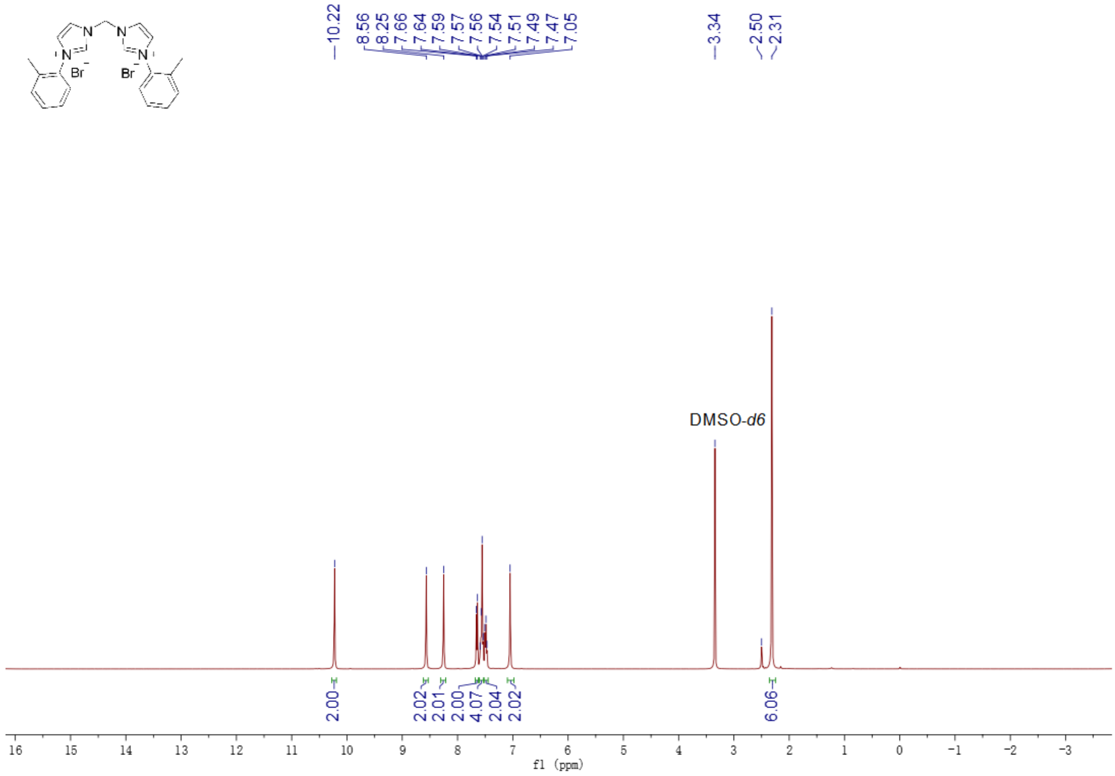

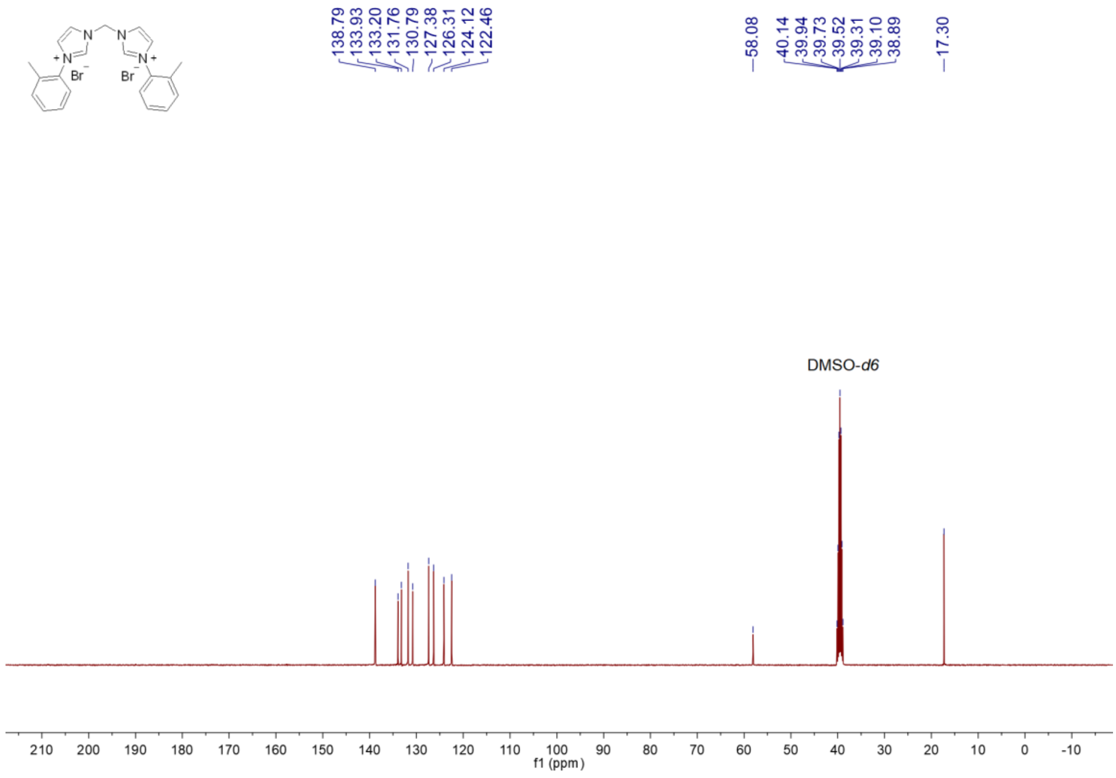

^{1}H NMR (400 MHz, DMSO-*d6*) and ^{13}C NMR (100 MHz, DMSO-*d6*) spectra of product L4

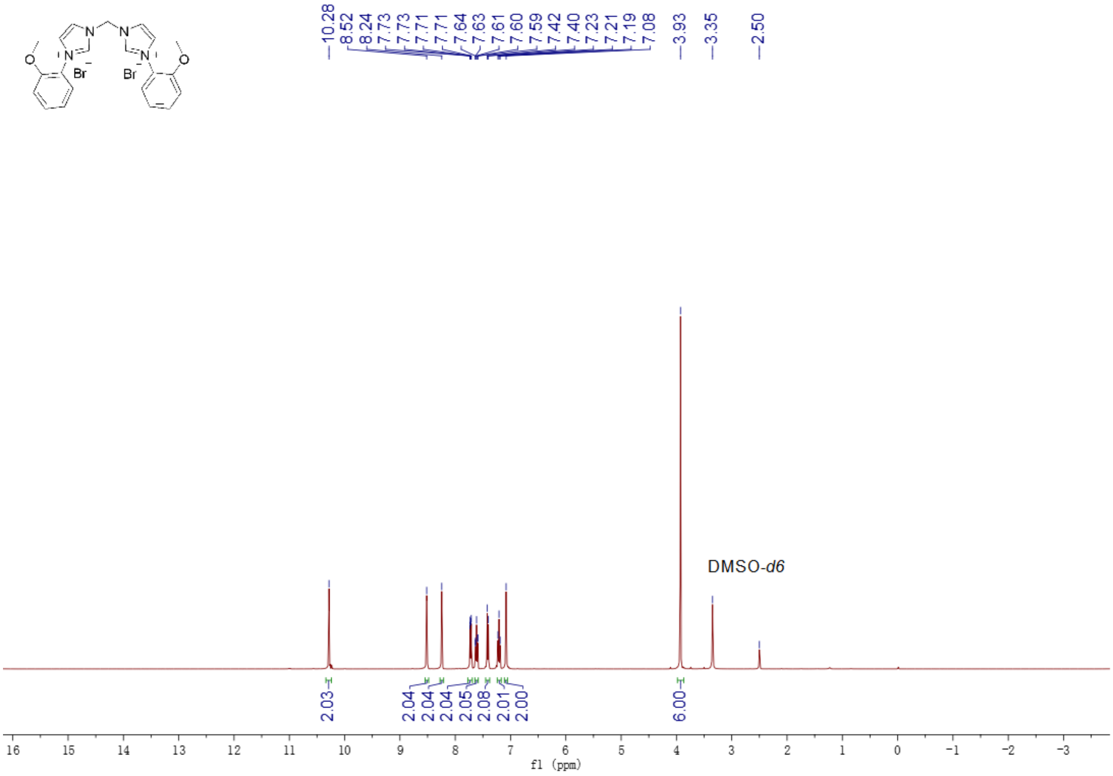

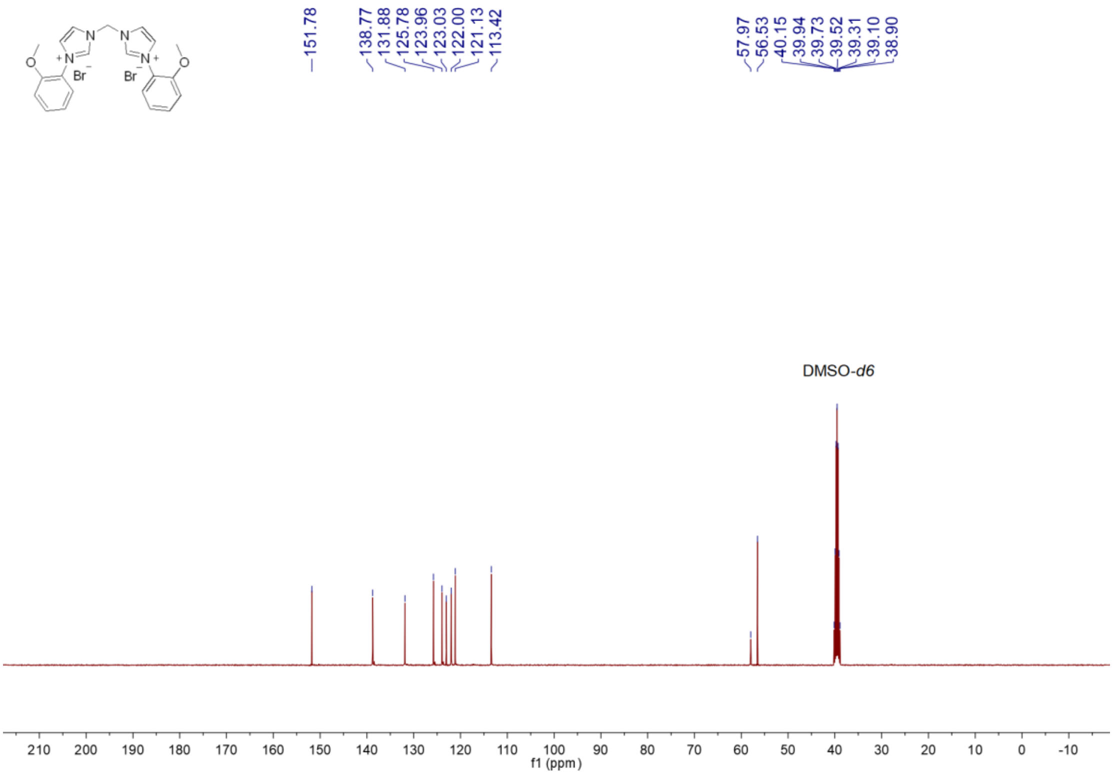

^{1}H NMR (400 MHz, DMSO-*d6*) and ^{13}C NMR (100 MHz, DMSO-*d6*) spectra of product L5

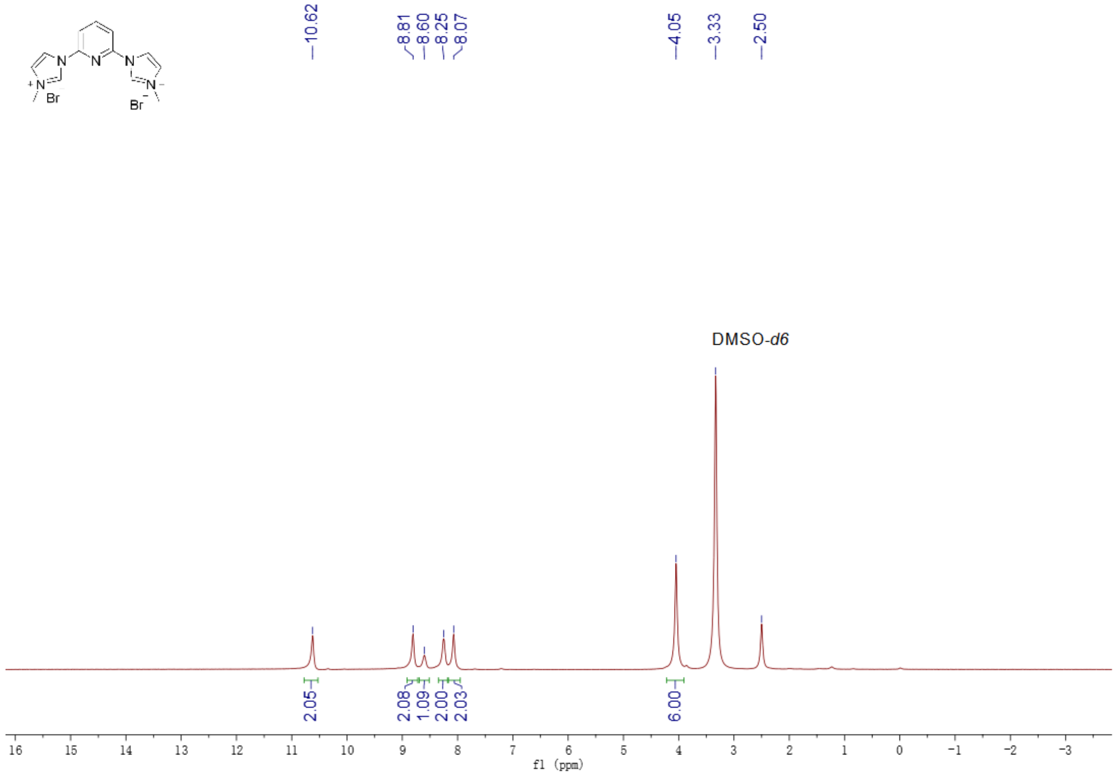

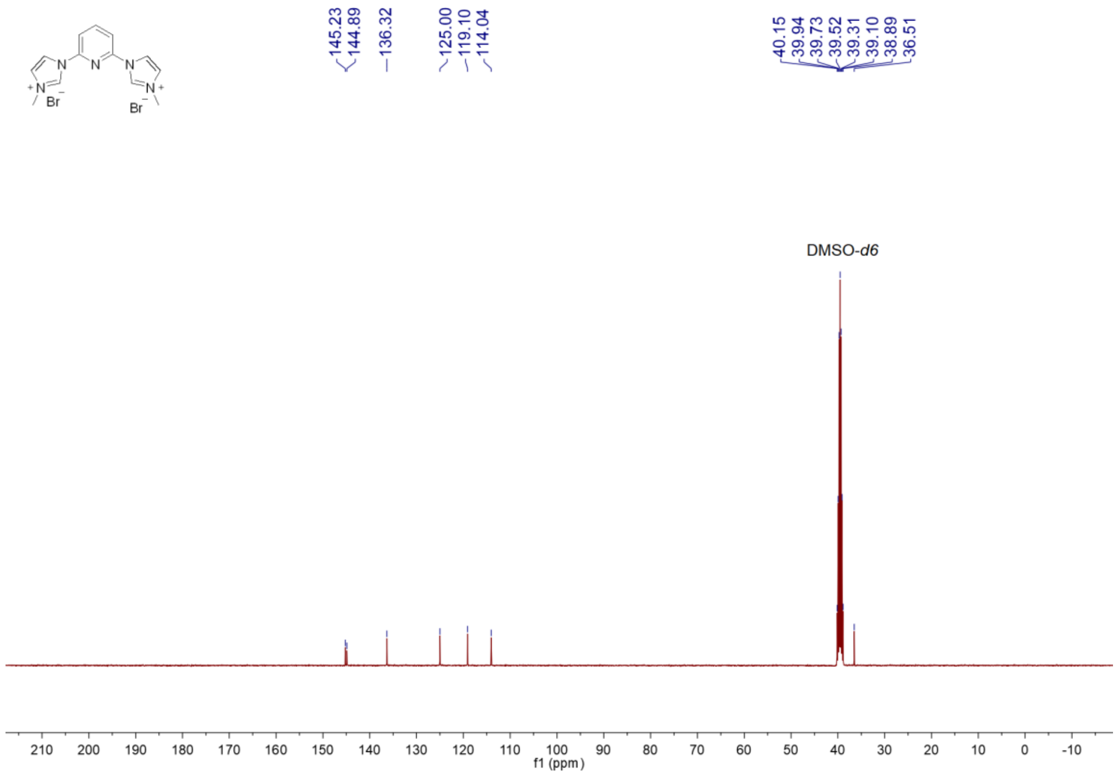

^{1}H NMR (400 MHz, DMSO-*d6*) and ^{13}C NMR (100 MHz, DMSO-*d6*) spectra of product L6

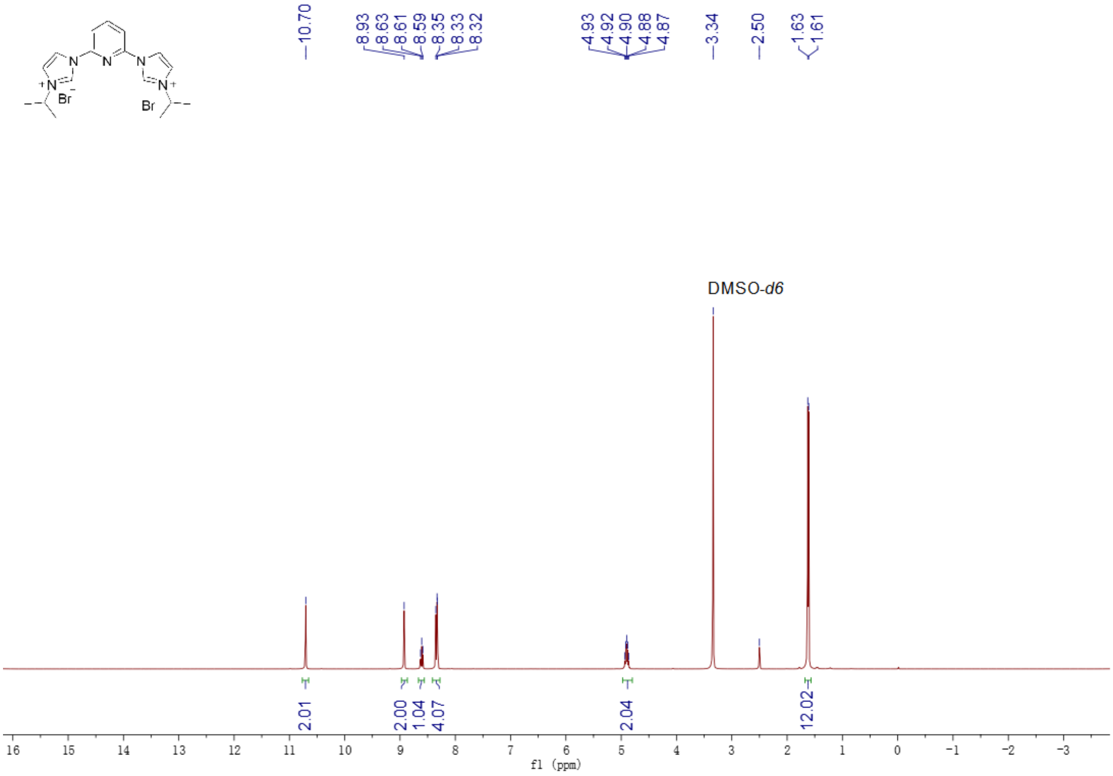

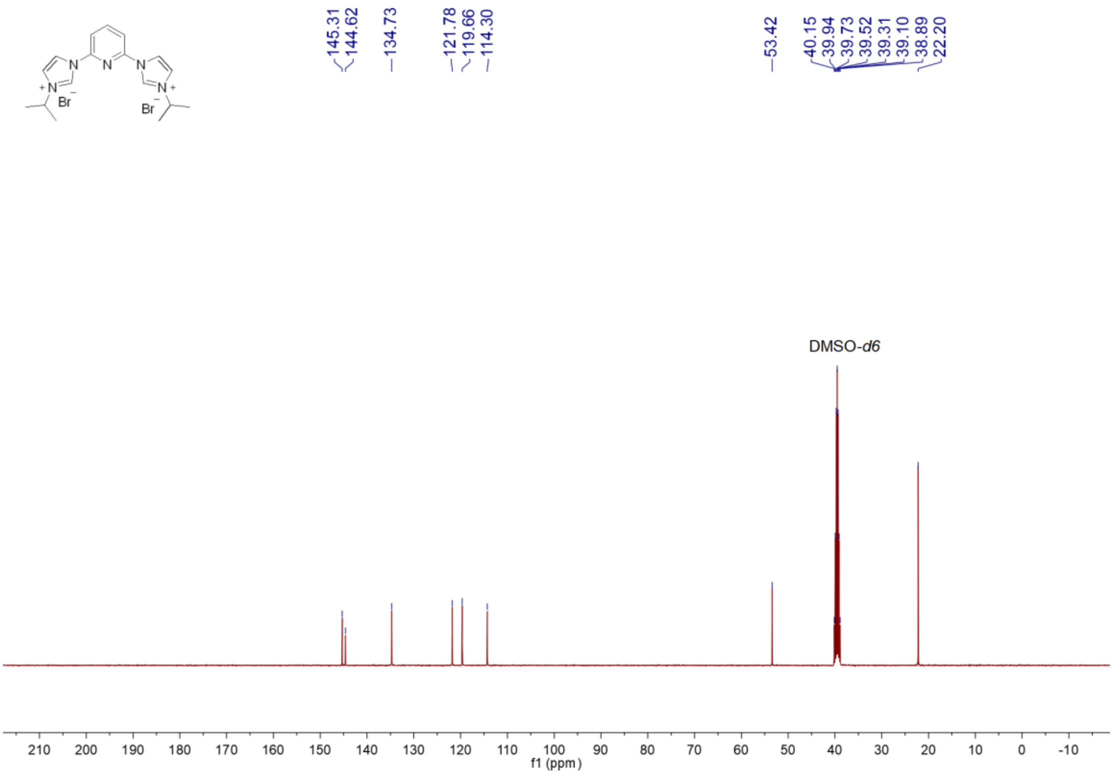

^{1}H NMR (400 MHz, $CDCl_3$) and ^{13}C NMR (100 MHz, $CDCl_3$) spectra of product 2a

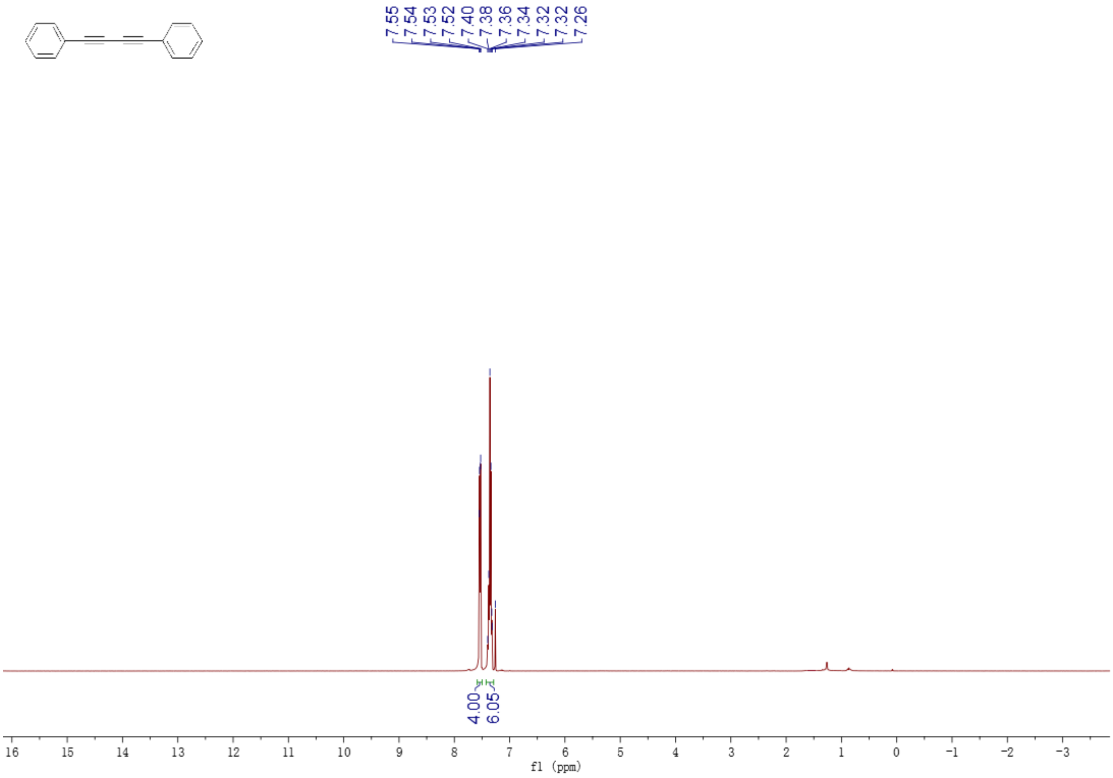

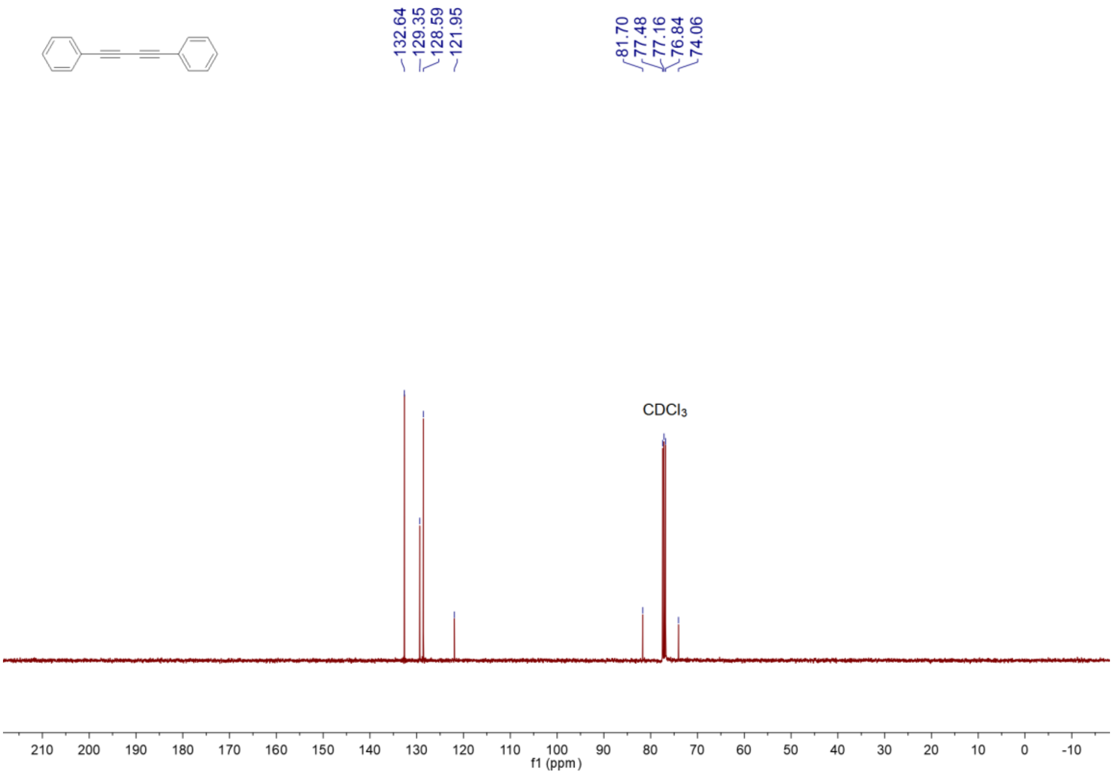

^{1}H NMR (400 MHz, $CDCl_3$) and ^{13}C NMR (100 MHz, $CDCl_3$) spectra of product 2b

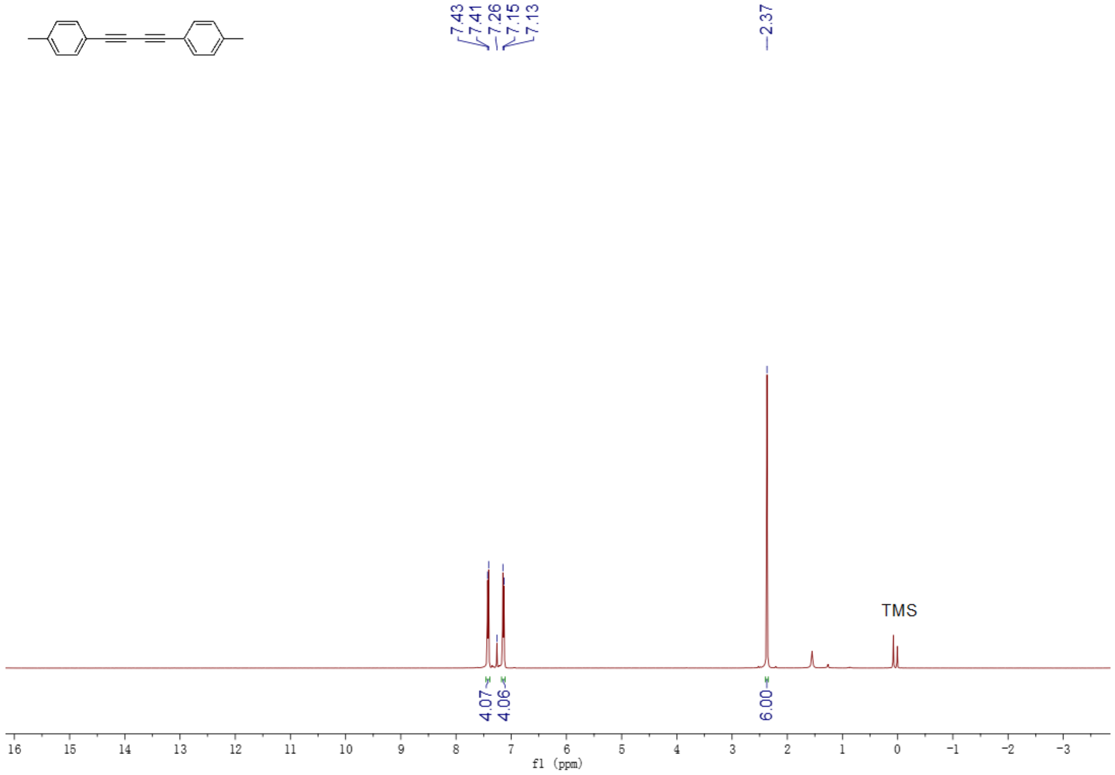

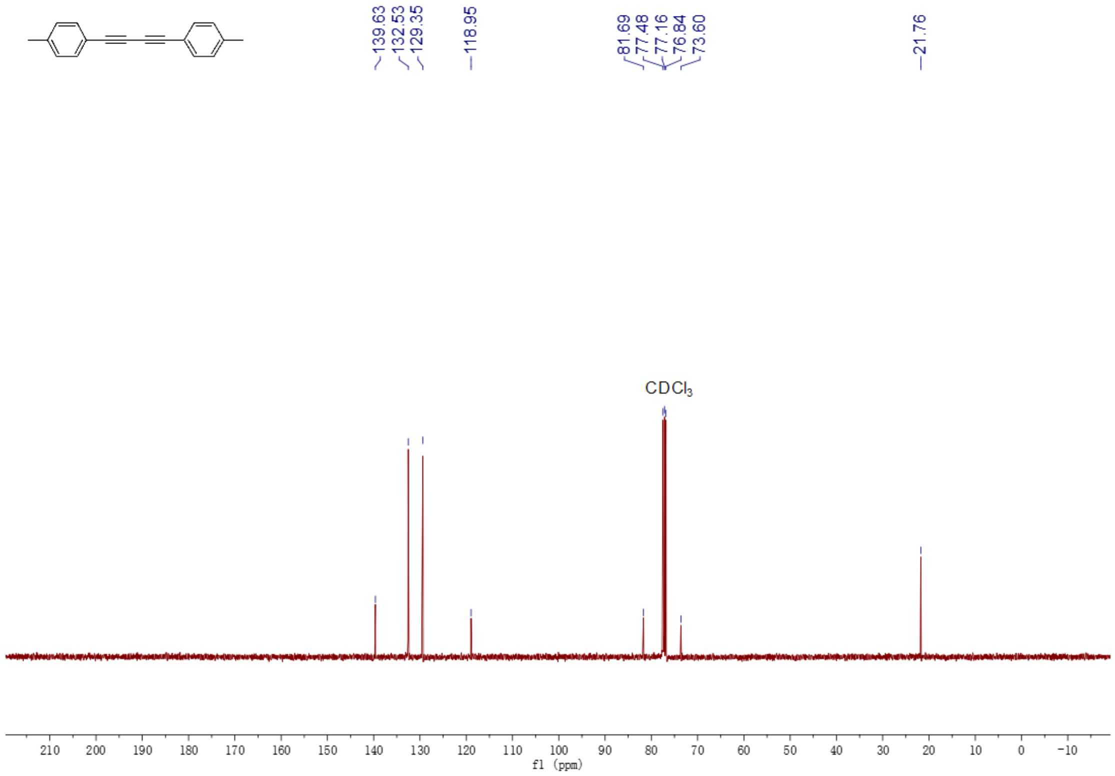

^{1}H NMR (400 MHz, $CDCl_3$) and ^{13}C NMR (100 MHz, $CDCl_3$) spectra of product 2c

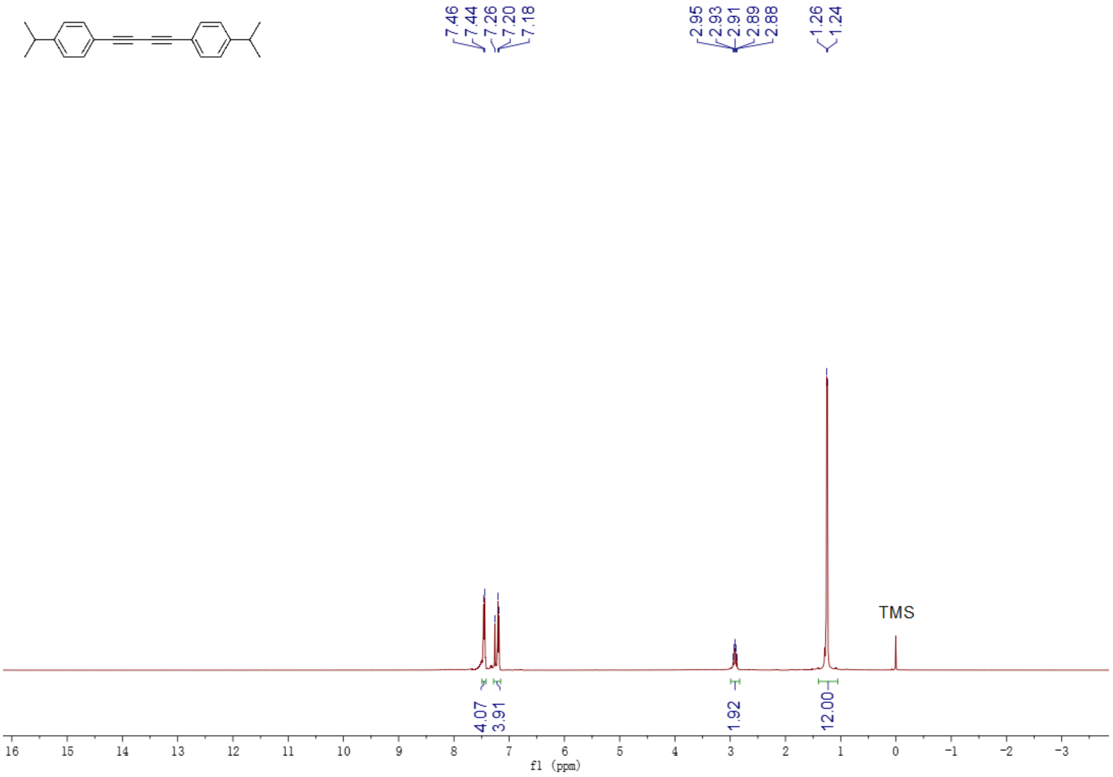

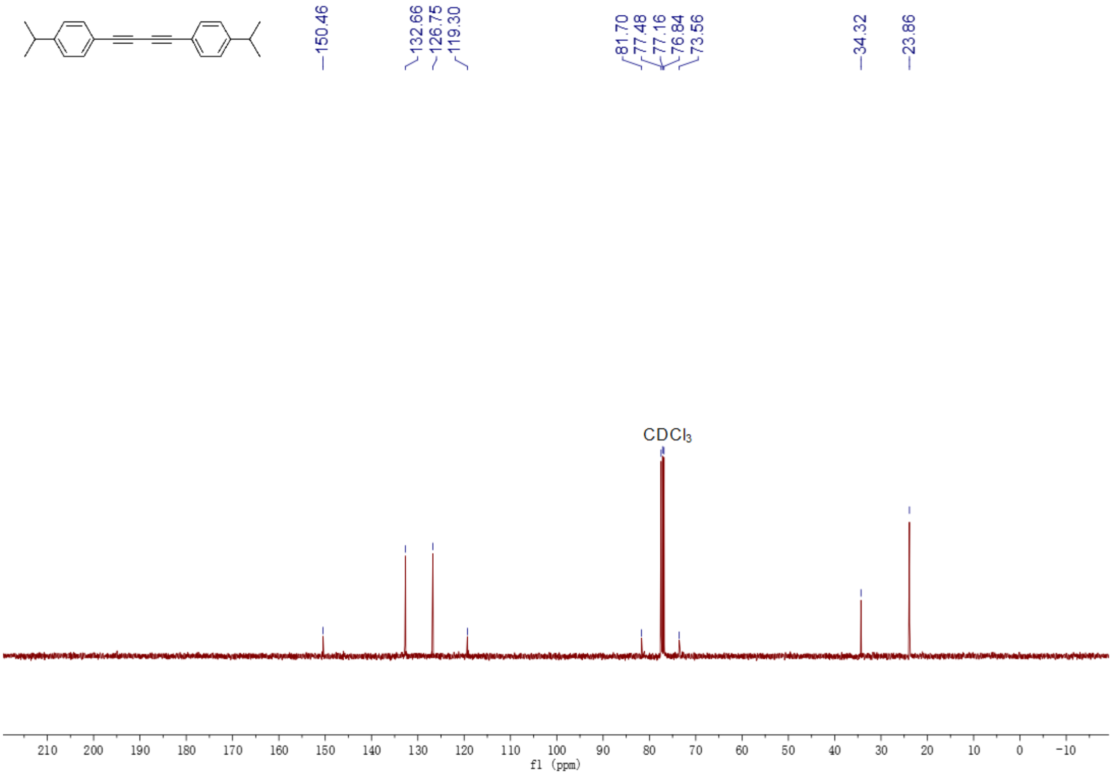

^{1}H NMR (400 MHz, $CDCl_3$) and ^{13}C NMR (100 MHz, $CDCl_3$) spectra of product **2d**

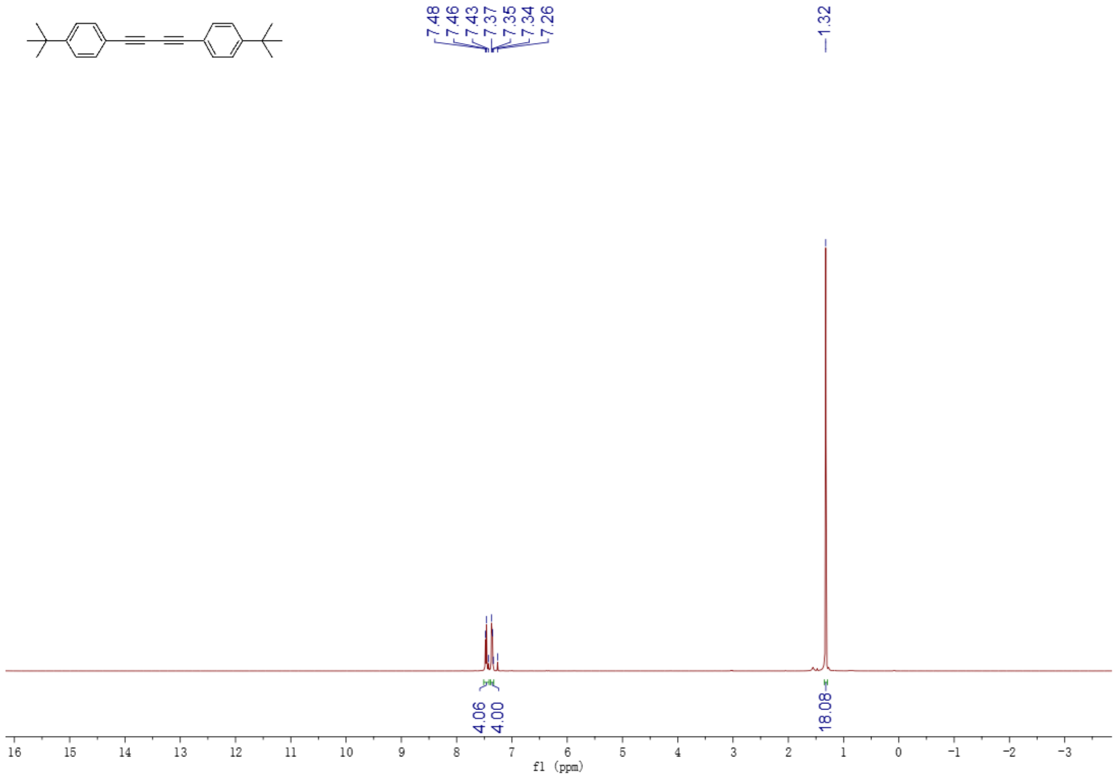

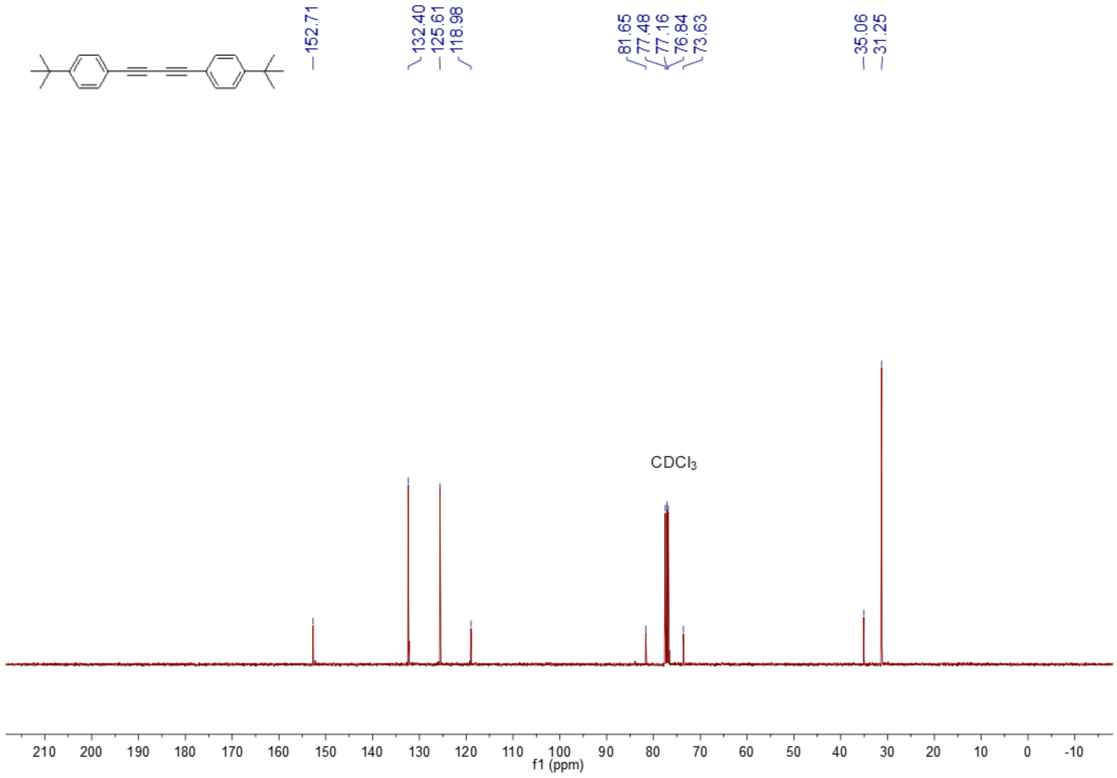

^{1}H NMR (400 MHz, $CDCl_3$) and ^{13}C NMR (100 MHz, $CDCl_3$) spectra of product **2e**

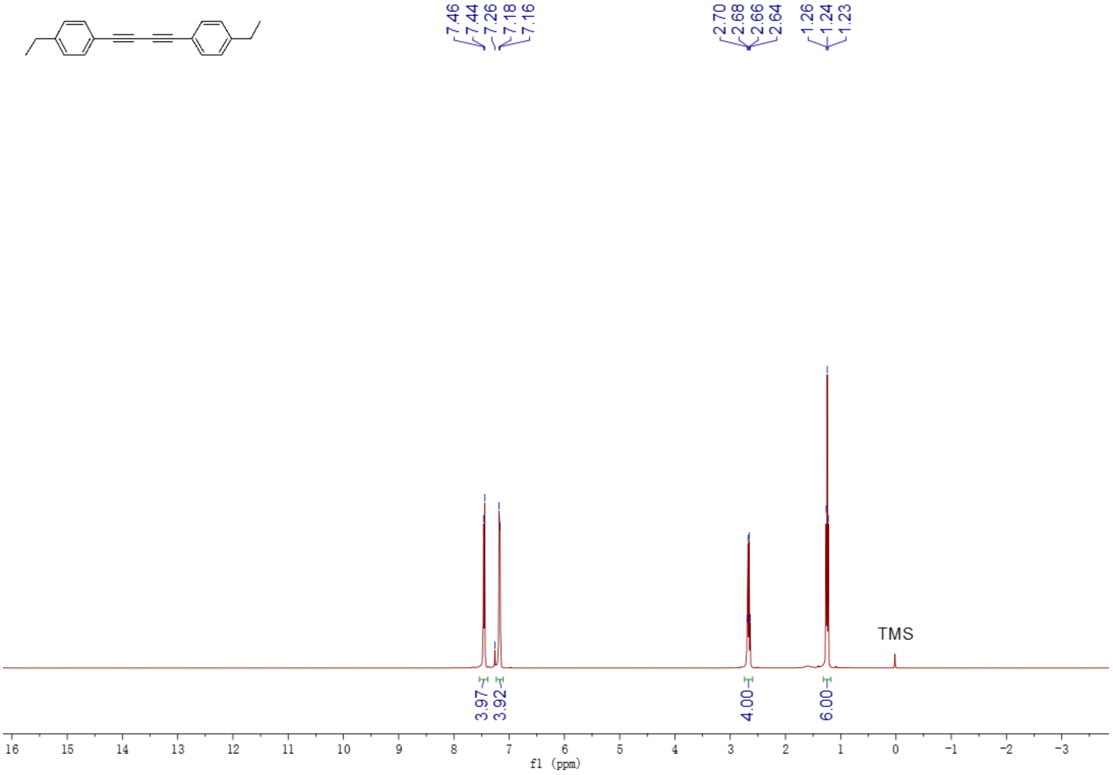

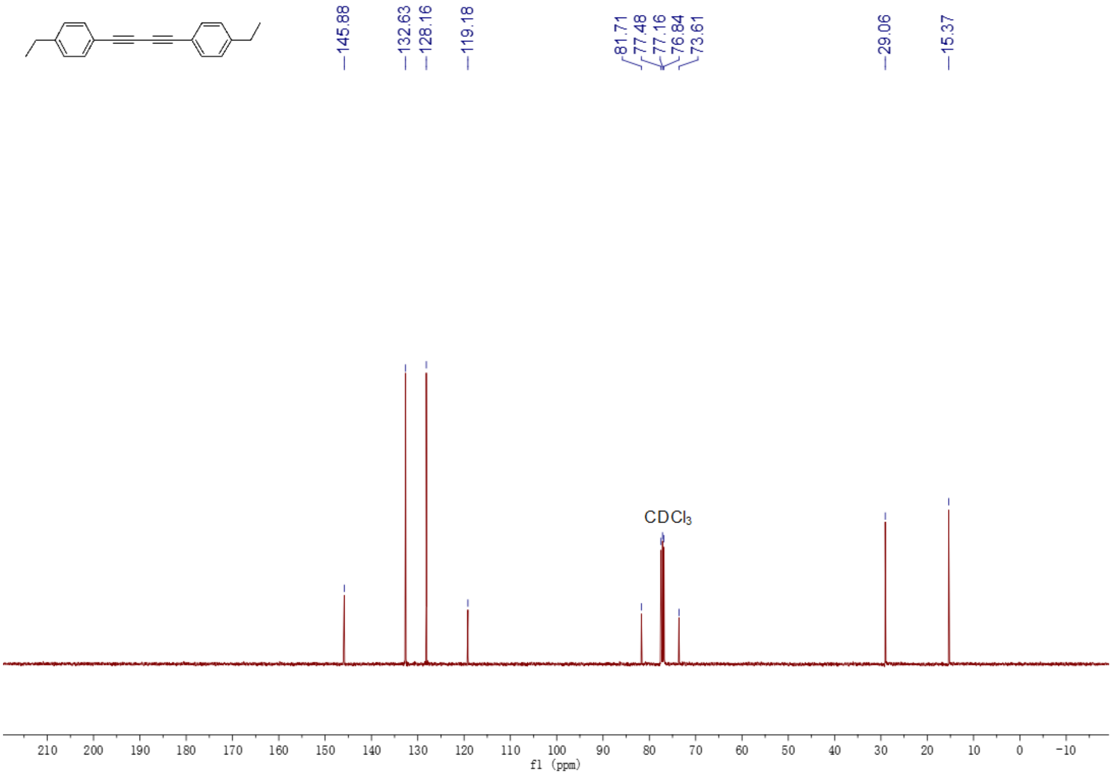

^1H NMR (400 MHz, $CDCl_3$) and ^{13}C NMR (100 MHz, $CDCl_3$) spectra of product **2f**

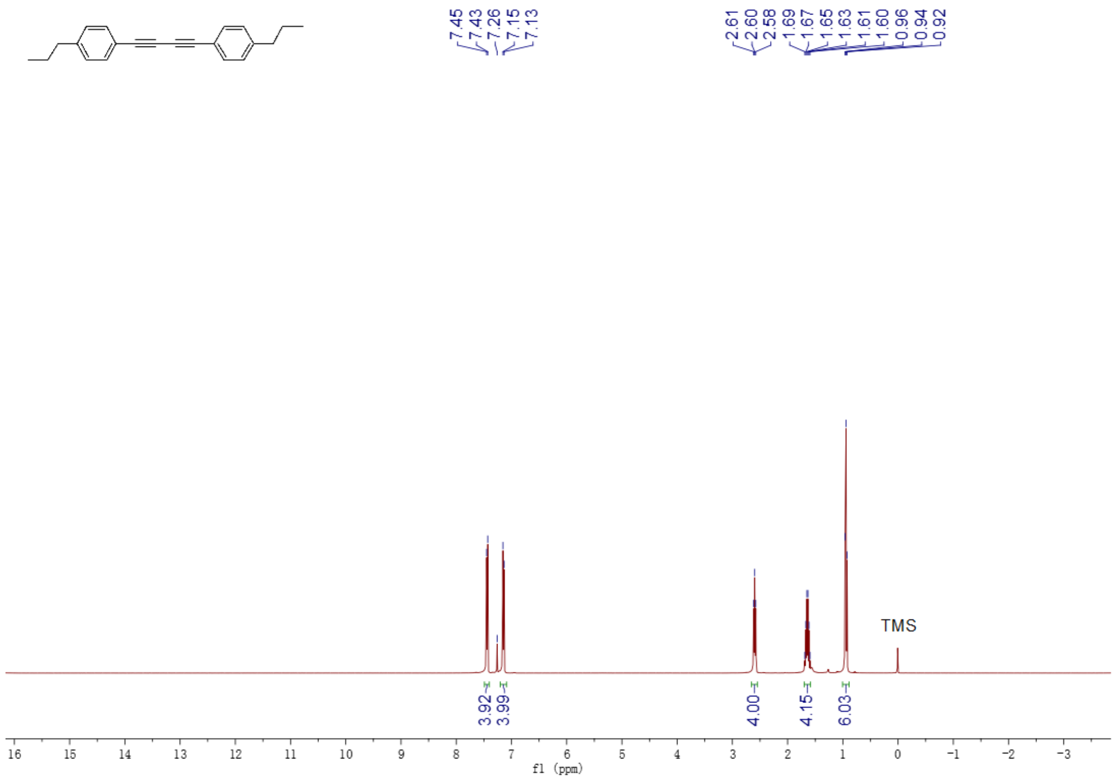

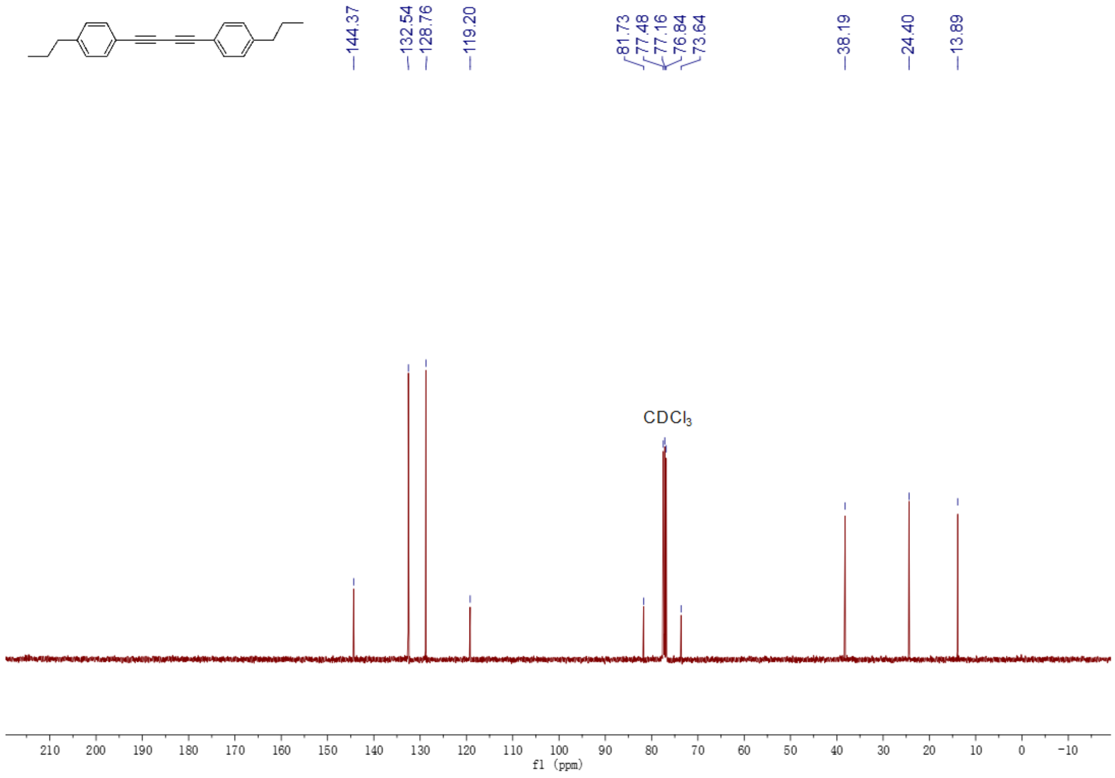

^1H NMR (400 MHz, $CDCl_3$) and ^{13}C NMR (100 MHz, $CDCl_3$) spectra of product **2g**

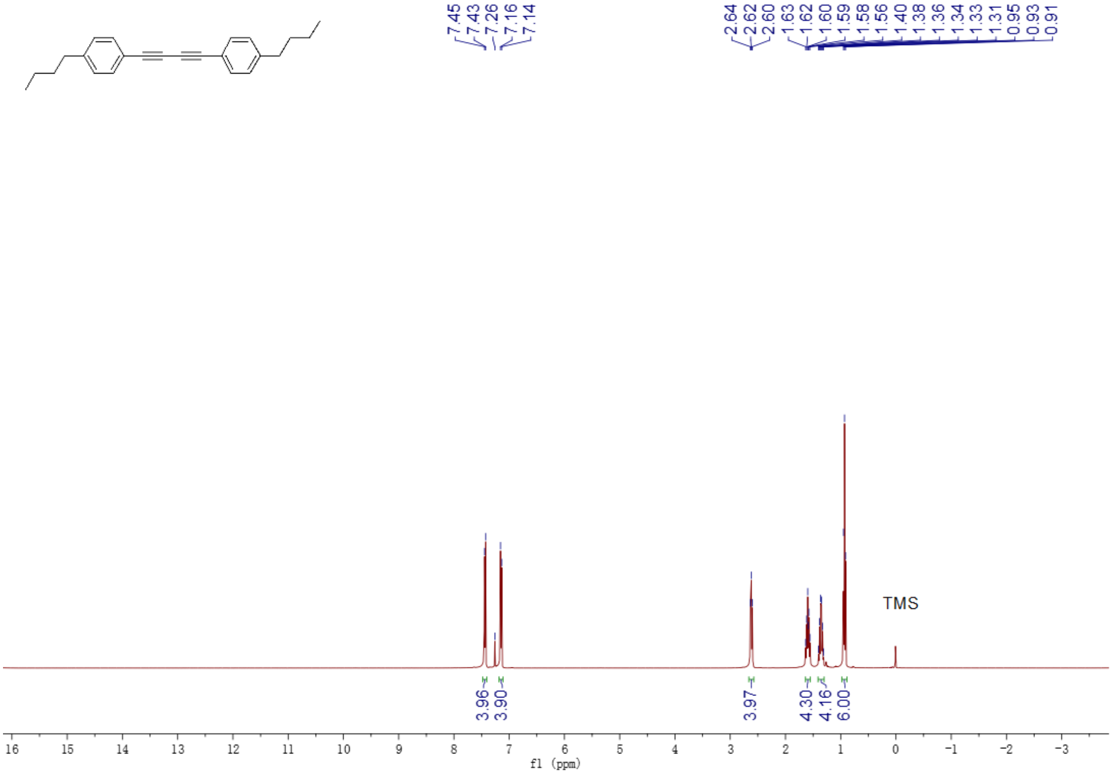

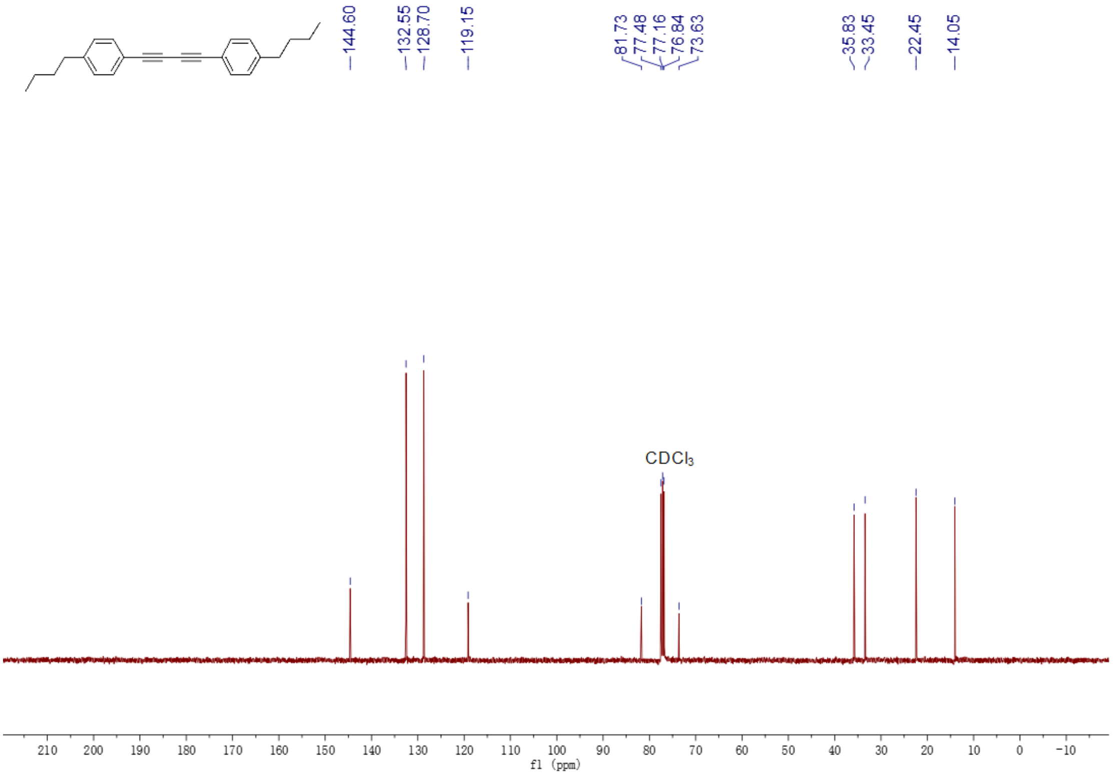

^{1}H NMR (400 MHz, $CDCl_3$) and ^{13}C NMR (100 MHz, $CDCl_3$) spectra of product **2h**

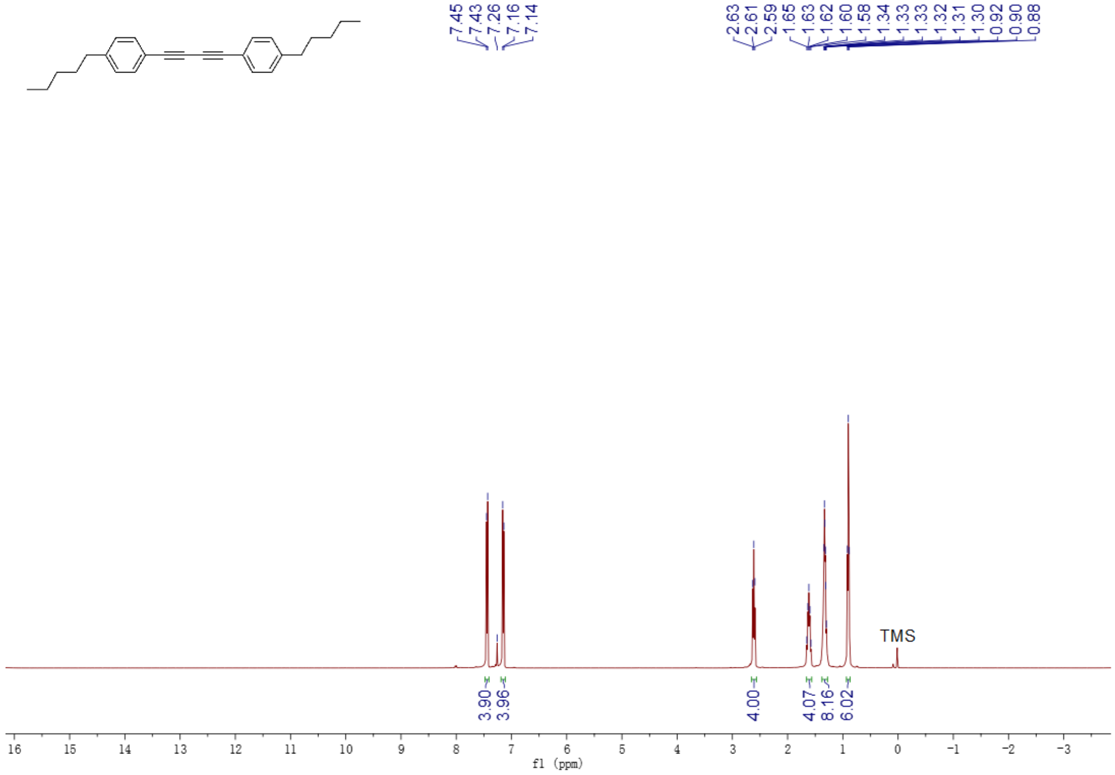

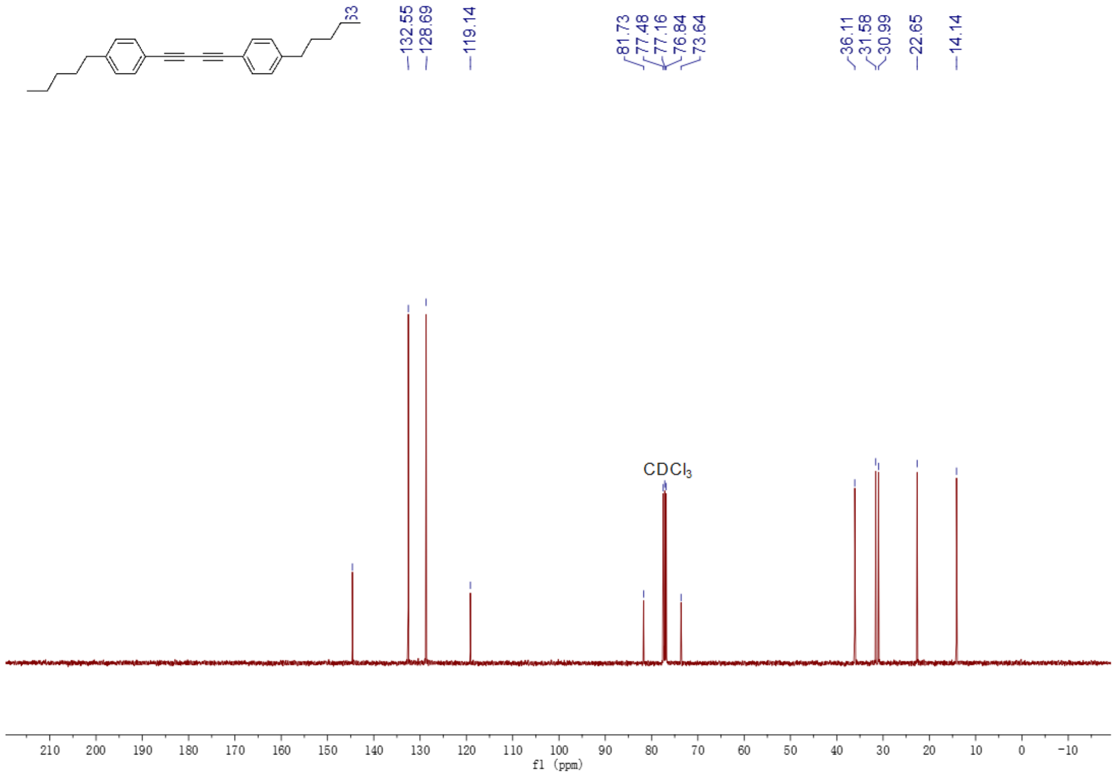

^{1}H NMR (400 MHz, $CDCl_3$) and ^{13}C NMR (100 MHz, $CDCl_3$) spectra of product 2i

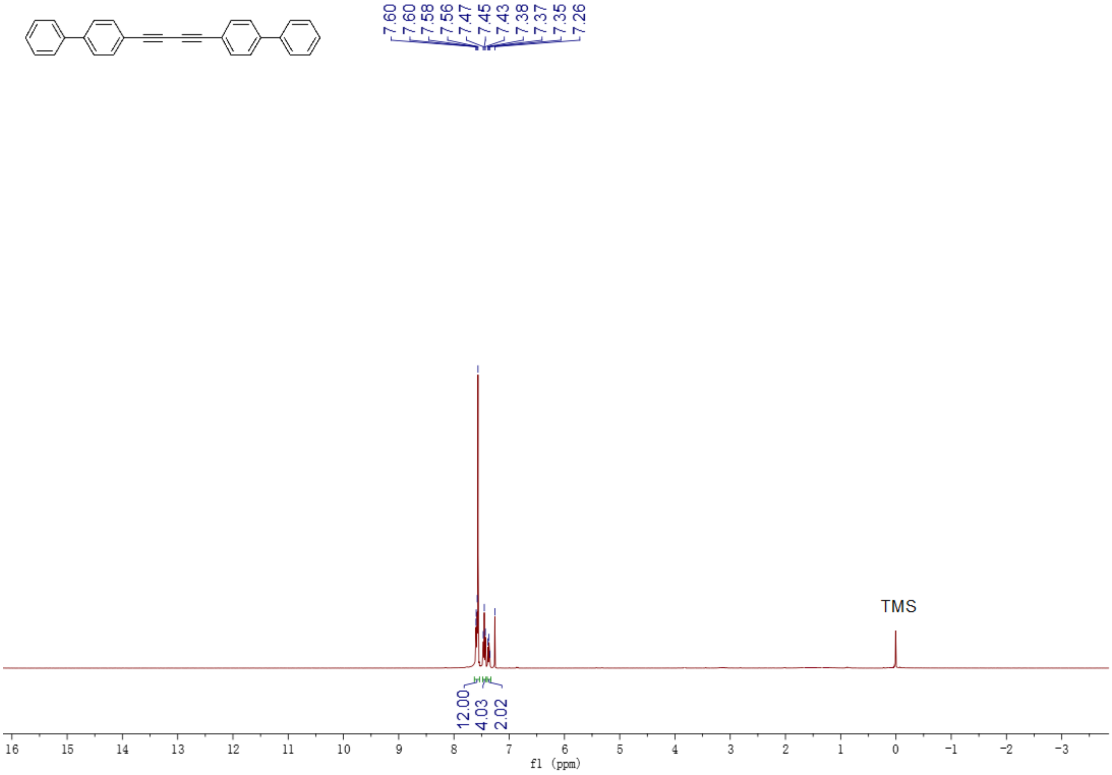

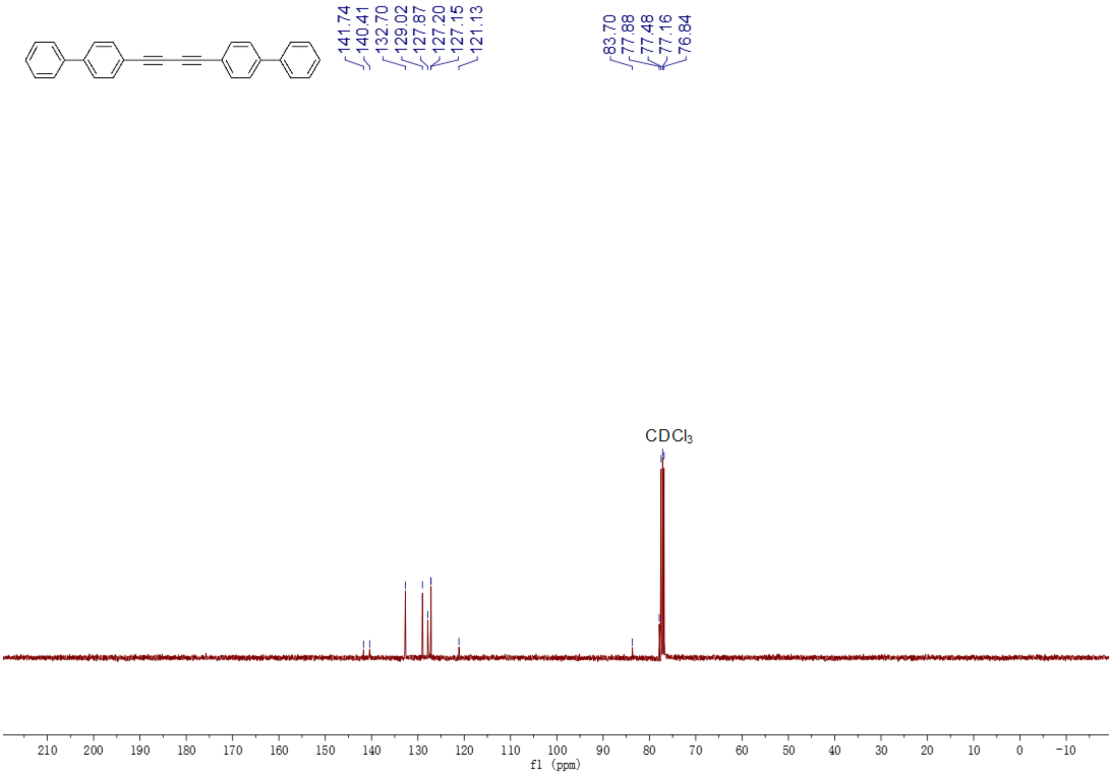

^{1}H NMR (400 MHz, $CDCl_3$) and ^{13}C NMR (100 MHz, $CDCl_3$) spectra of product 2j

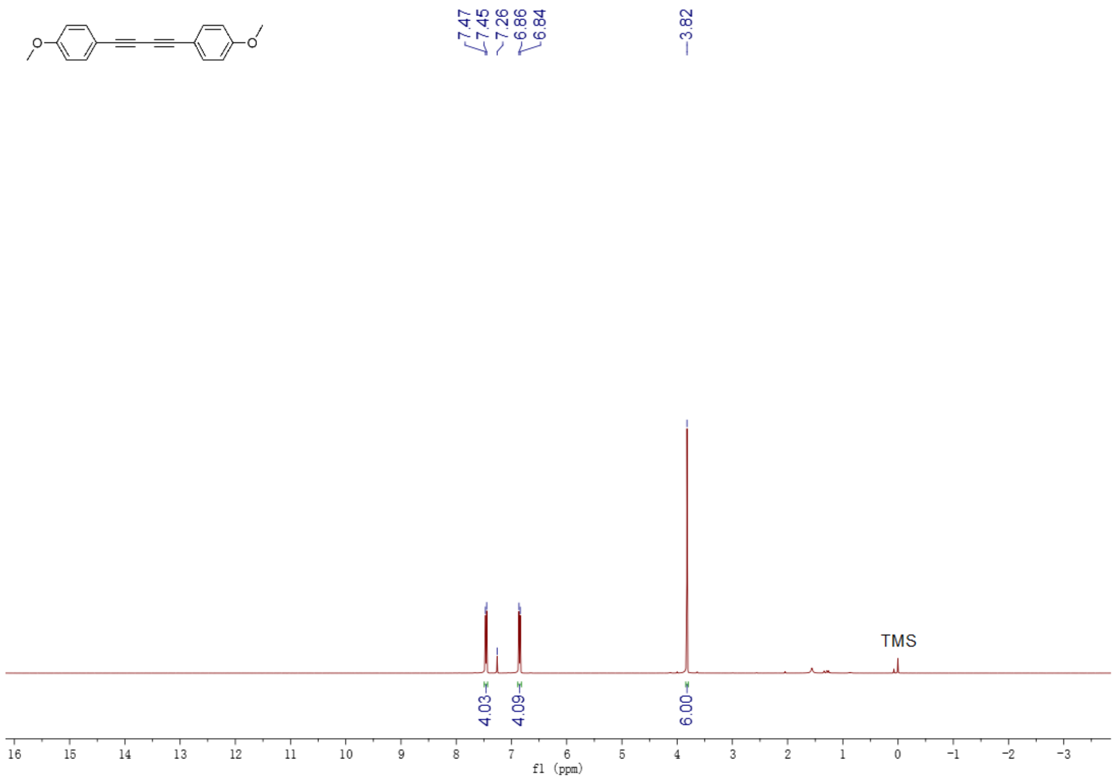

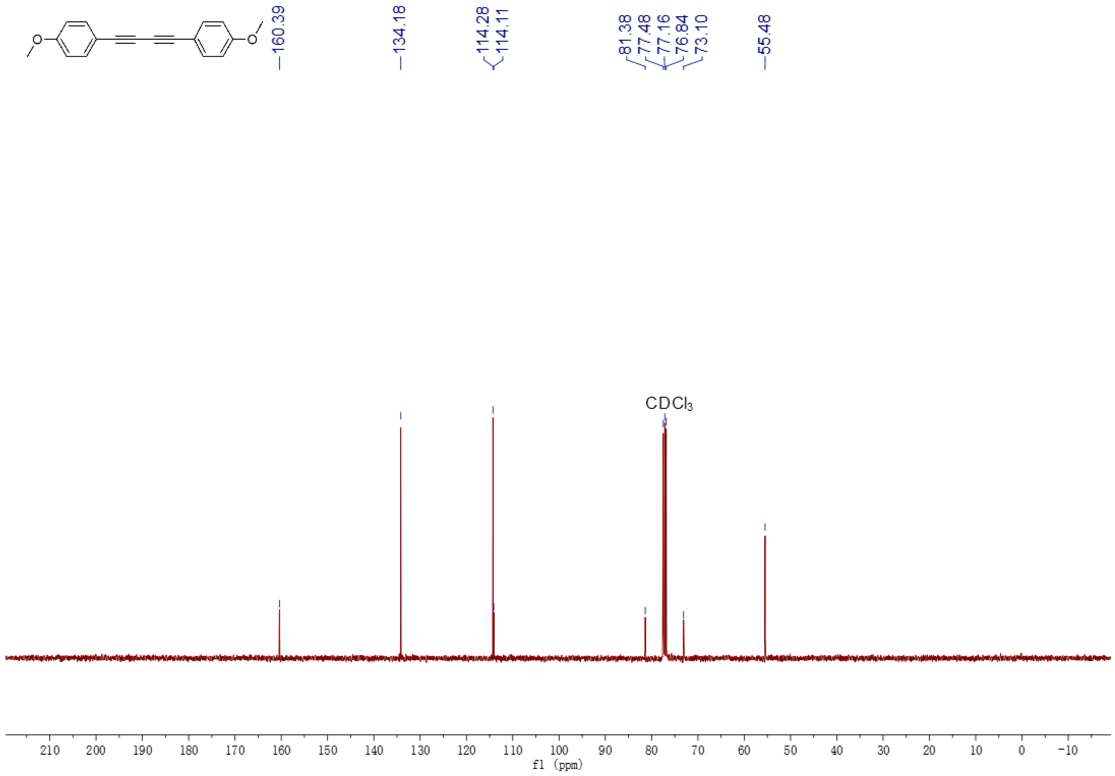

^{1}H NMR (400 MHz, $CDCl_3$) and ^{13}C NMR (100 MHz, $CDCl_3$) spectra of product **2k**

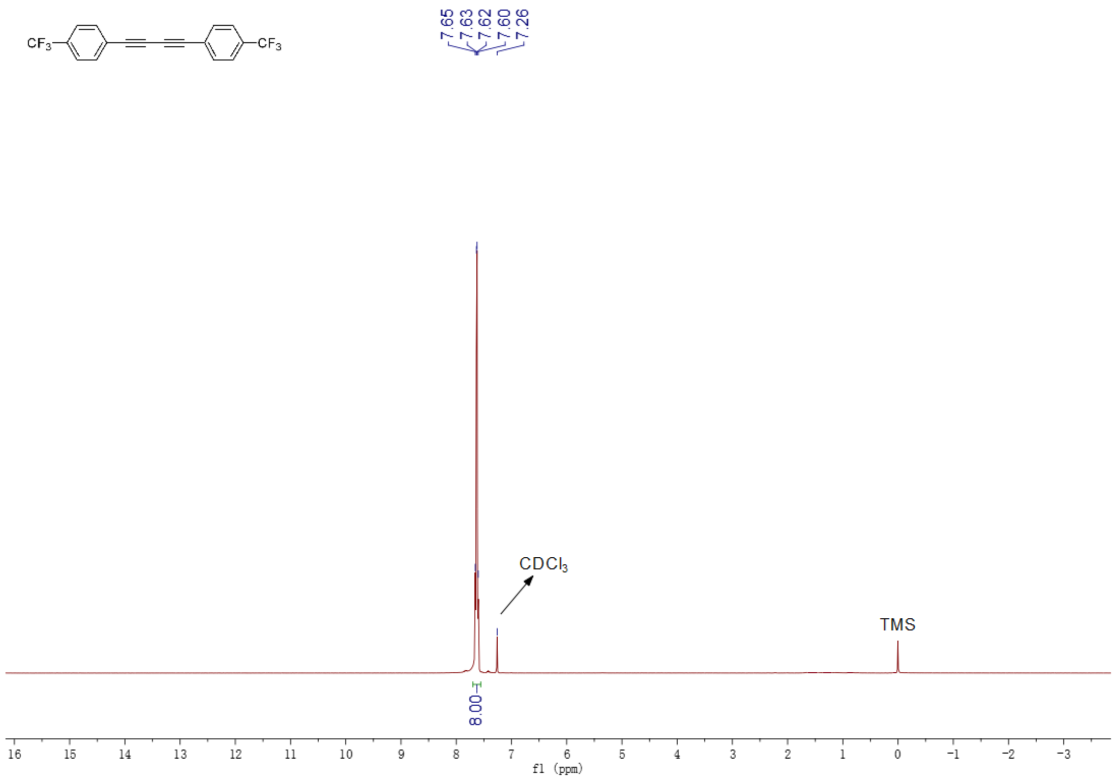

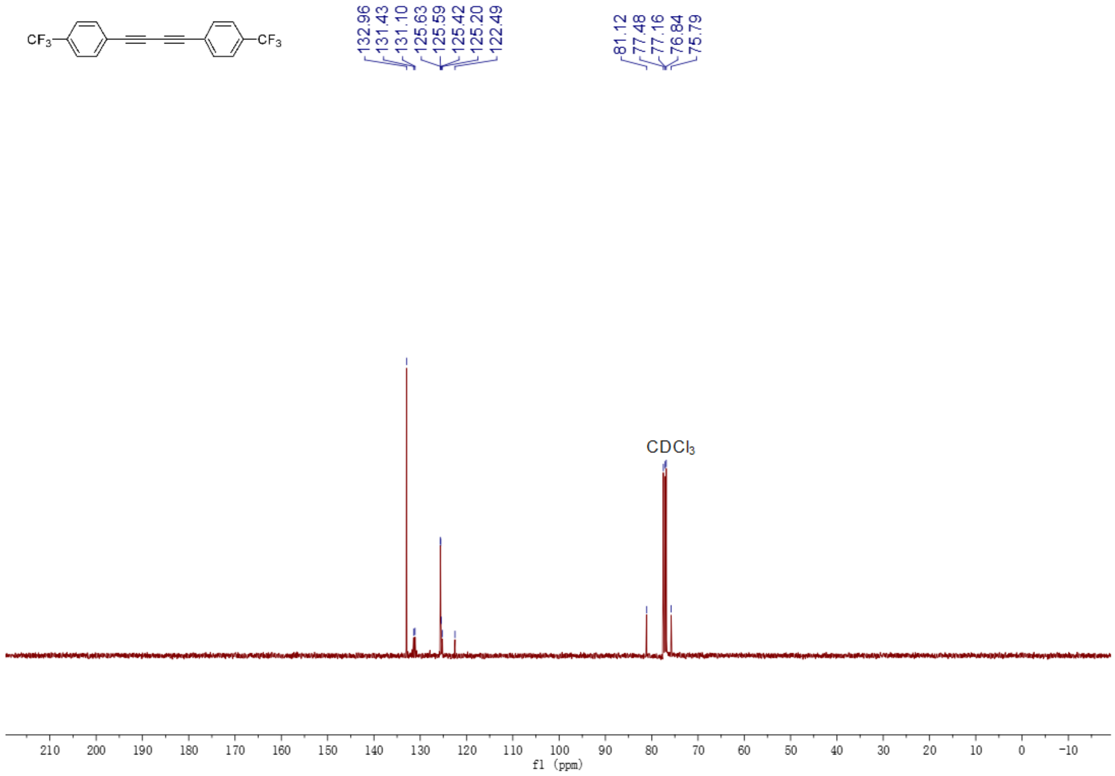

^{1}H NMR (400 MHz, $CDCl_3$) and ^{13}C NMR (100 MHz, $CDCl_3$) spectra of product 2l

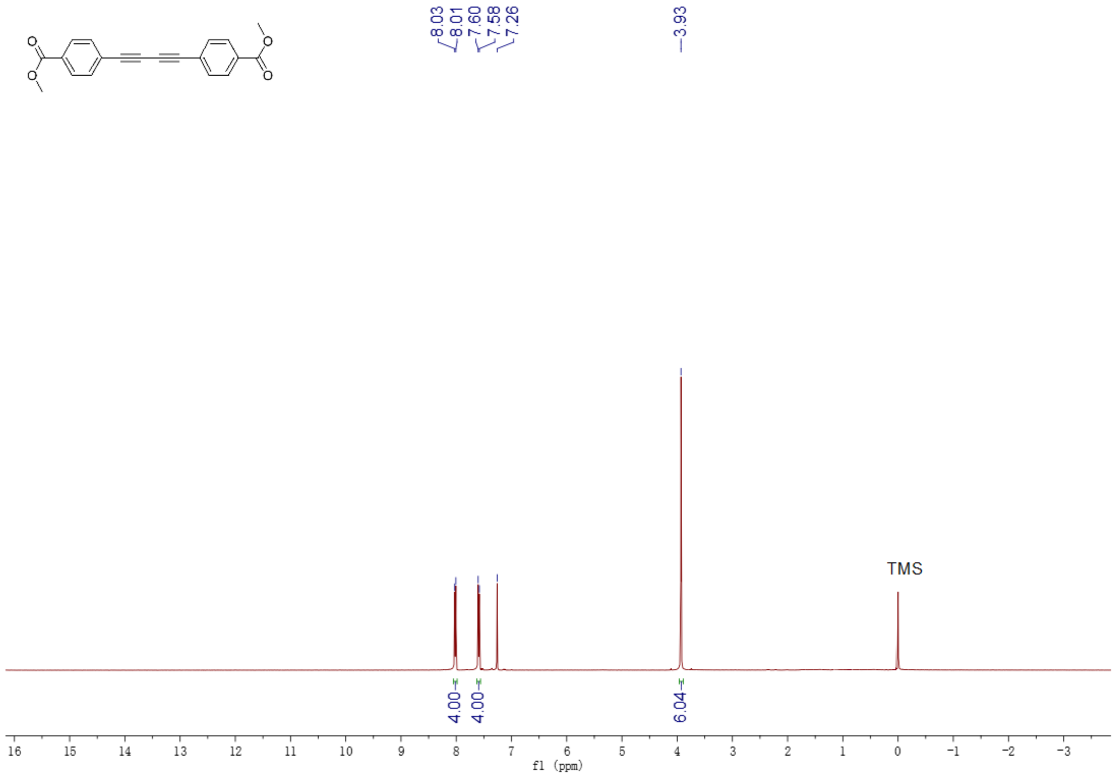

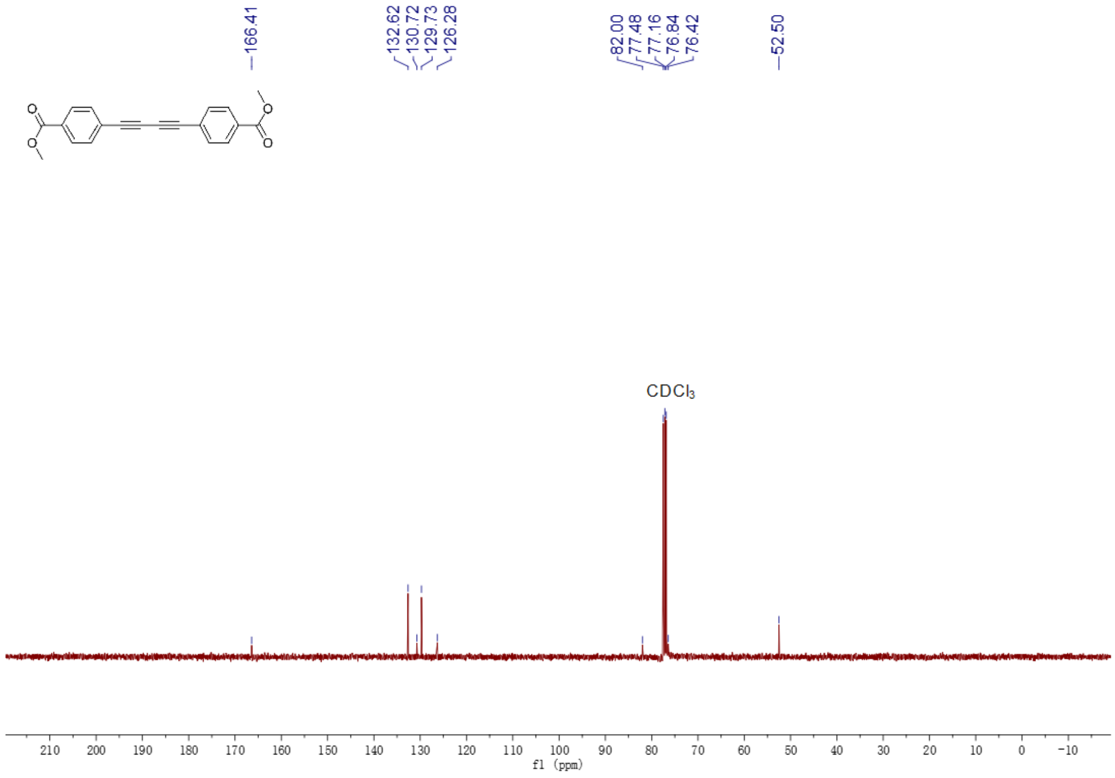

^{1}H NMR (400 MHz, $CDCl_3$) and ^{13}C NMR (100 MHz, $CDCl_3$) spectra of product **2m**

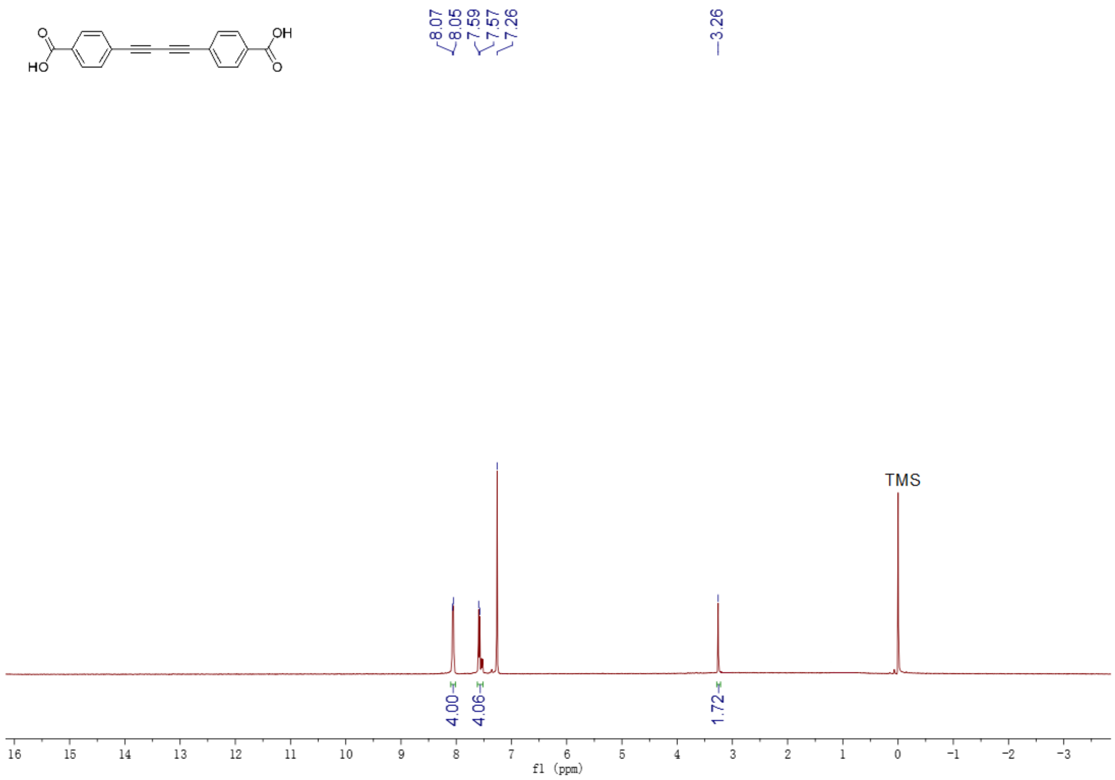

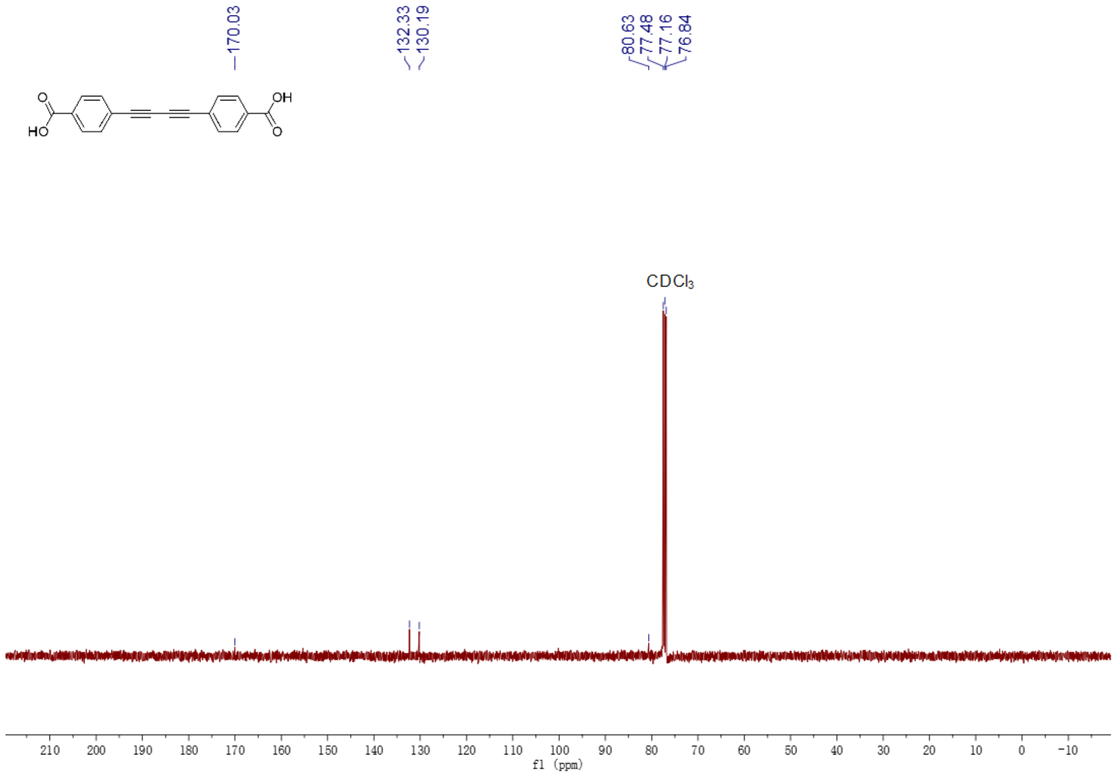

^{1}H NMR (400 MHz, $CDCl_3$) and ^{13}C NMR (100 MHz, $CDCl_3$) spectra of product 2n

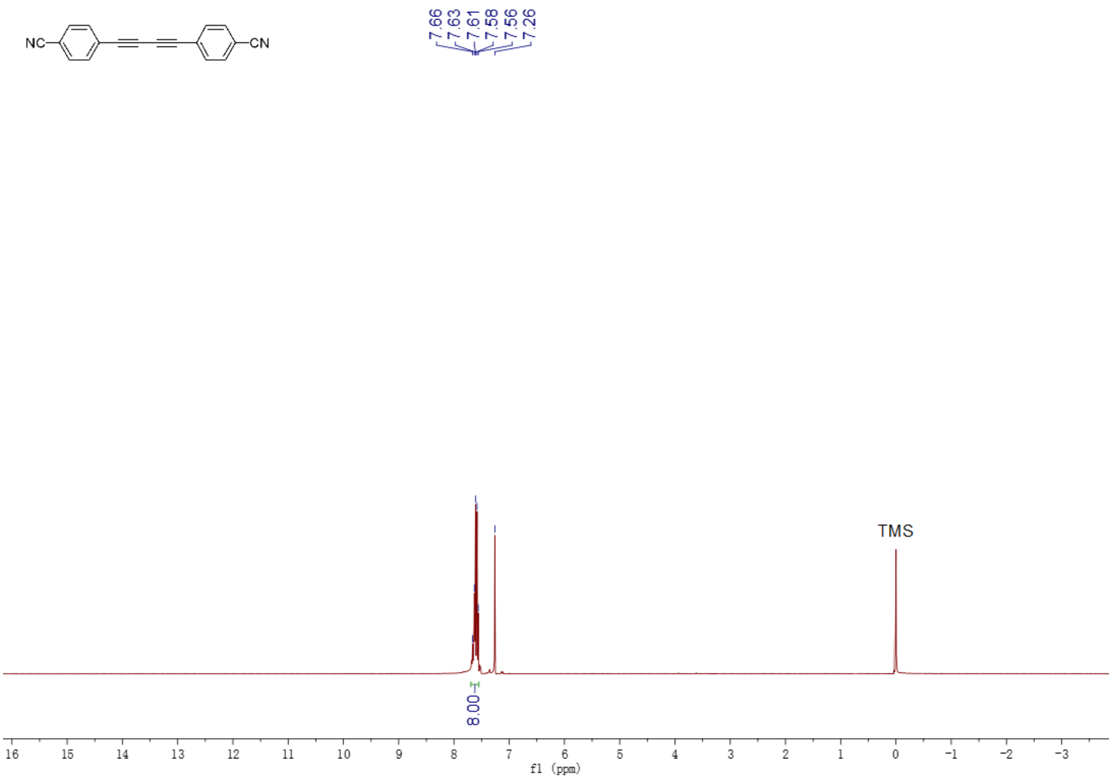

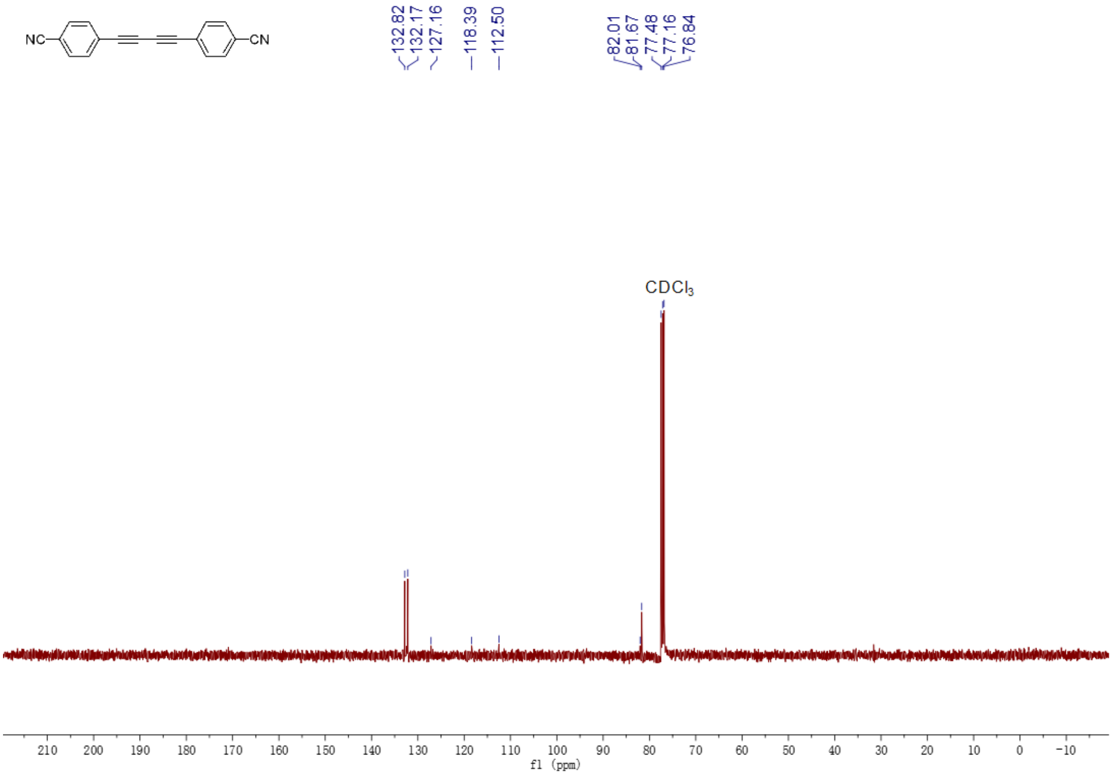

^{1}H NMR (400 MHz, $CDCl_3$) and ^{13}C NMR (100 MHz, $CDCl_3$) spectra of product **2o**

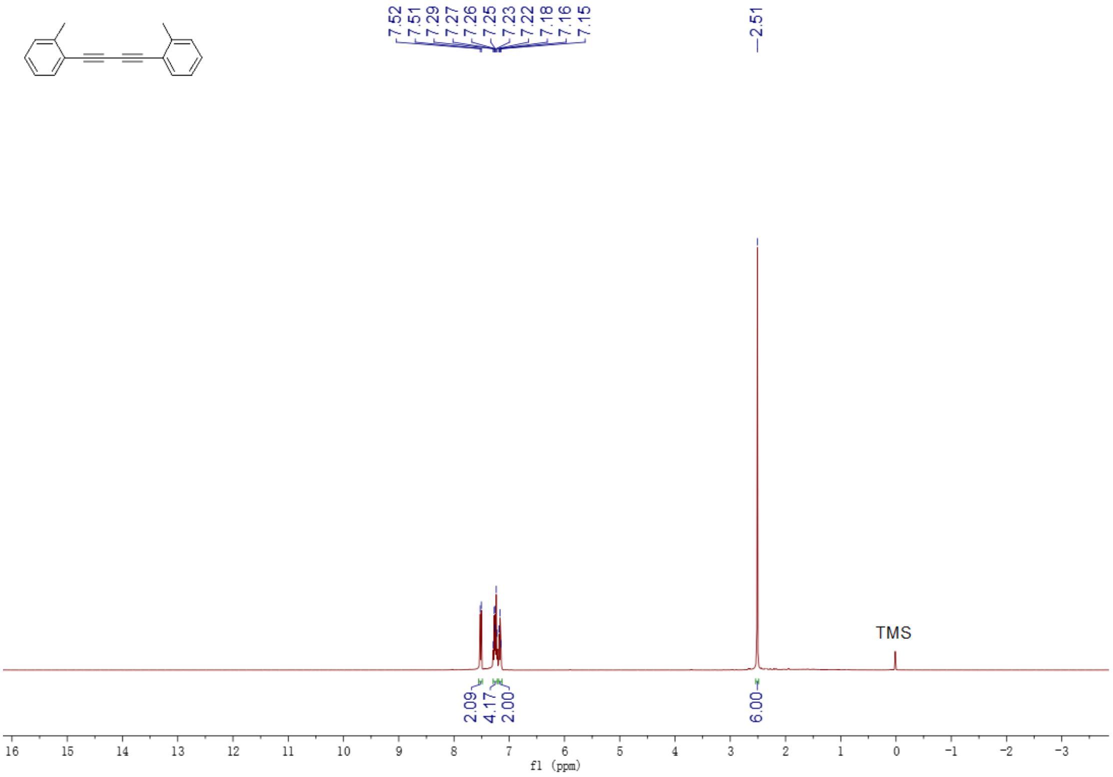

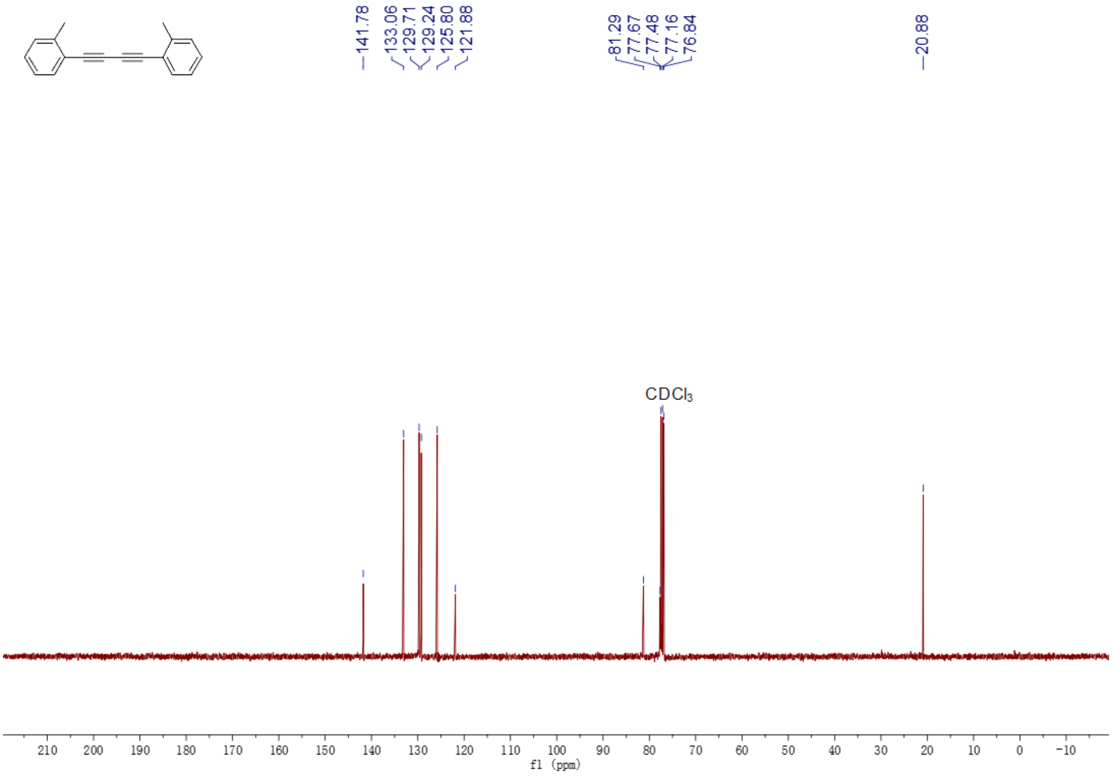

^{1}H NMR (400 MHz, $CDCl_3$) and ^{13}C NMR (100 MHz, $CDCl_3$) spectra of product 2p

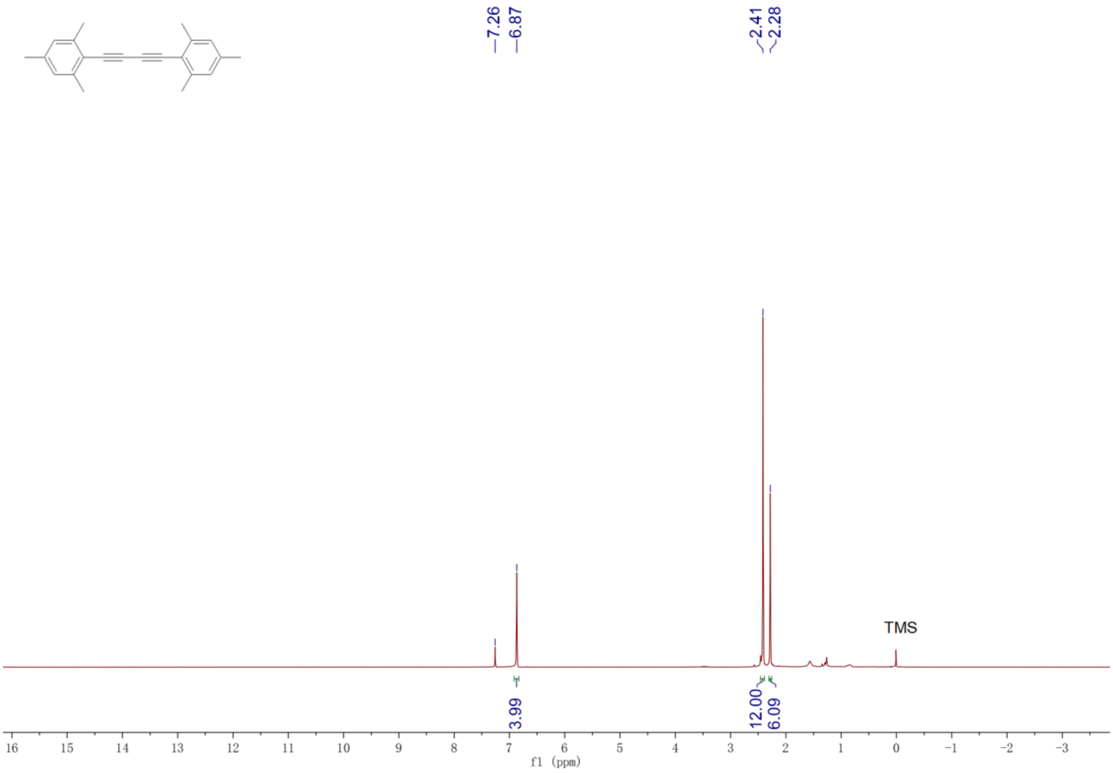

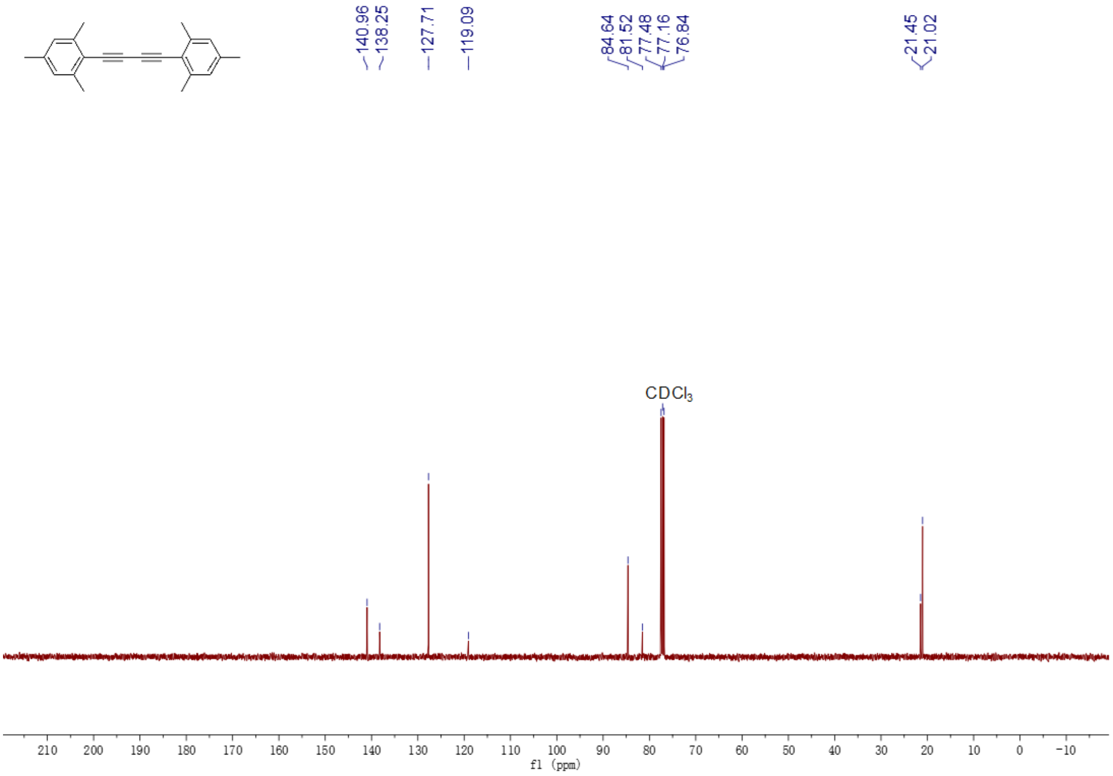

^{1}H NMR (400 MHz, $CDCl_3$) and ^{13}C NMR (100 MHz, $CDCl_3$) spectra of product 2q

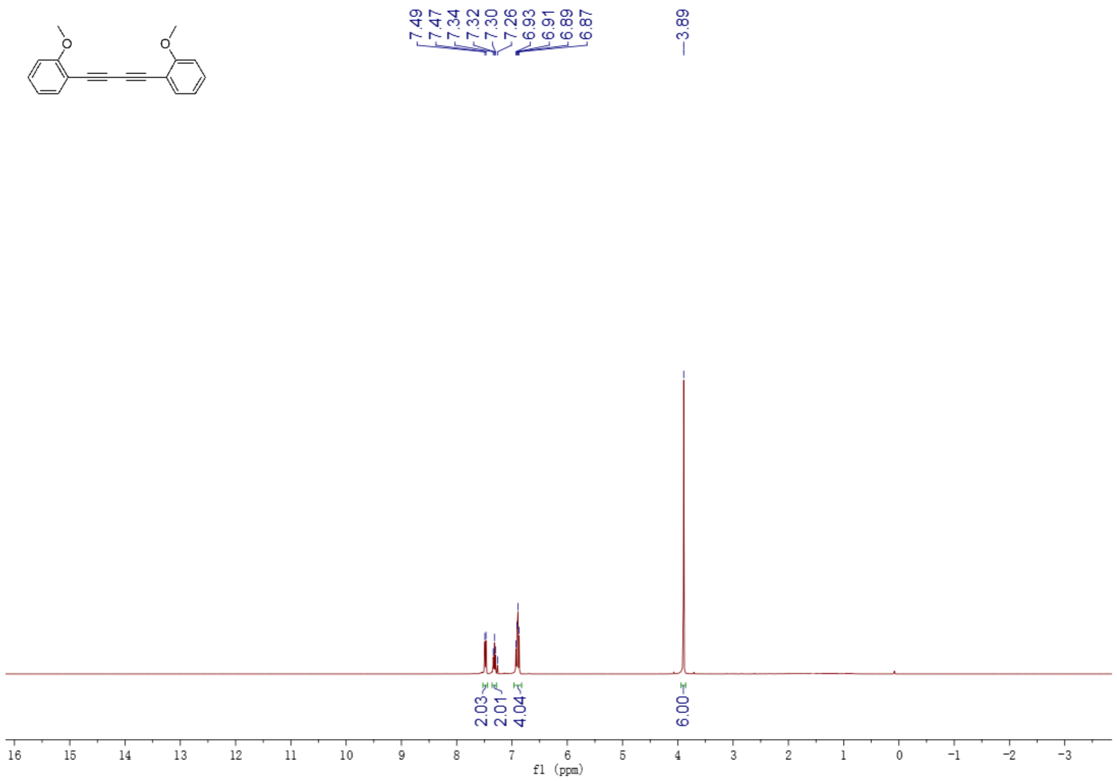

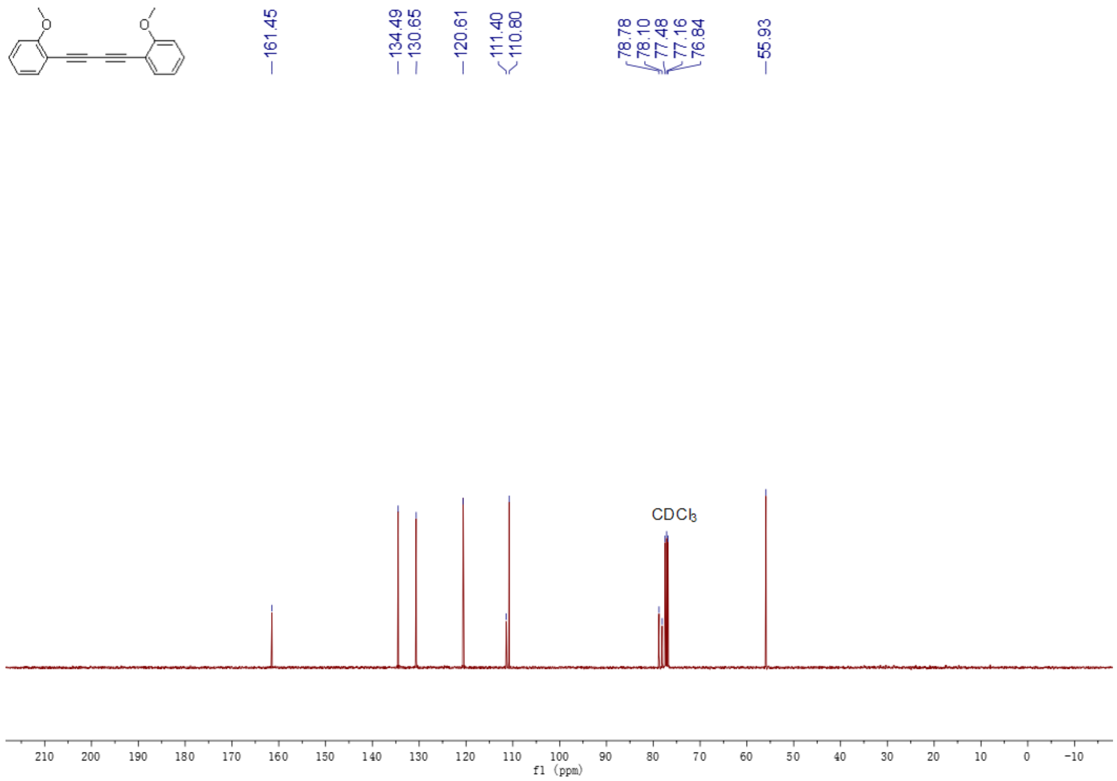

^{1}H NMR (400 MHz, $CDCl_3$) and ^{13}C NMR (100 MHz, $CDCl_3$) spectra of product 2r

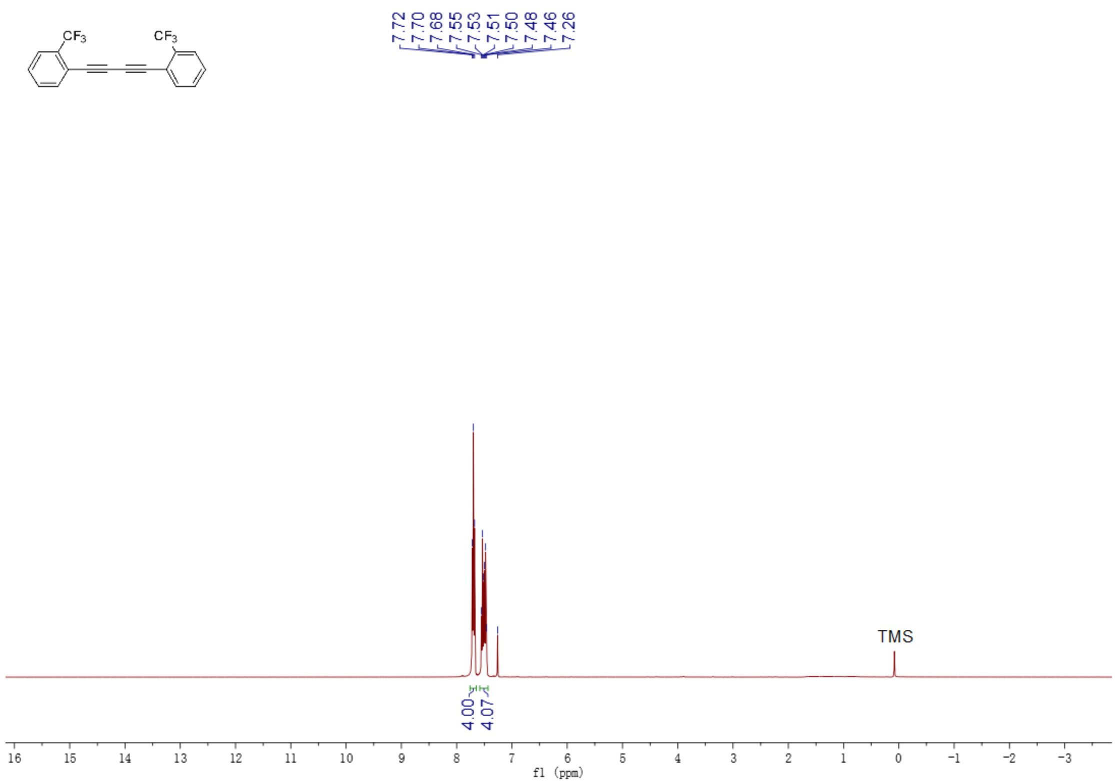

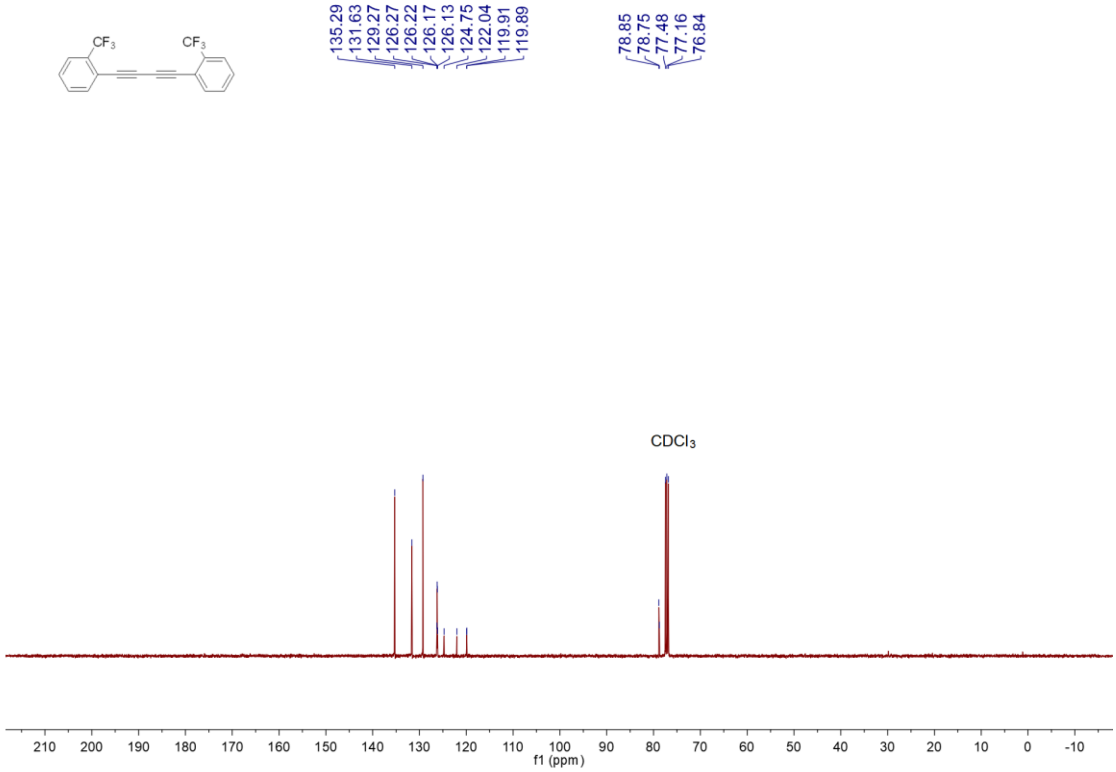

^{1}H NMR (400 MHz, $CDCl_3$) and ^{13}C NMR (100 MHz, $CDCl_3$) spectra of product 4a

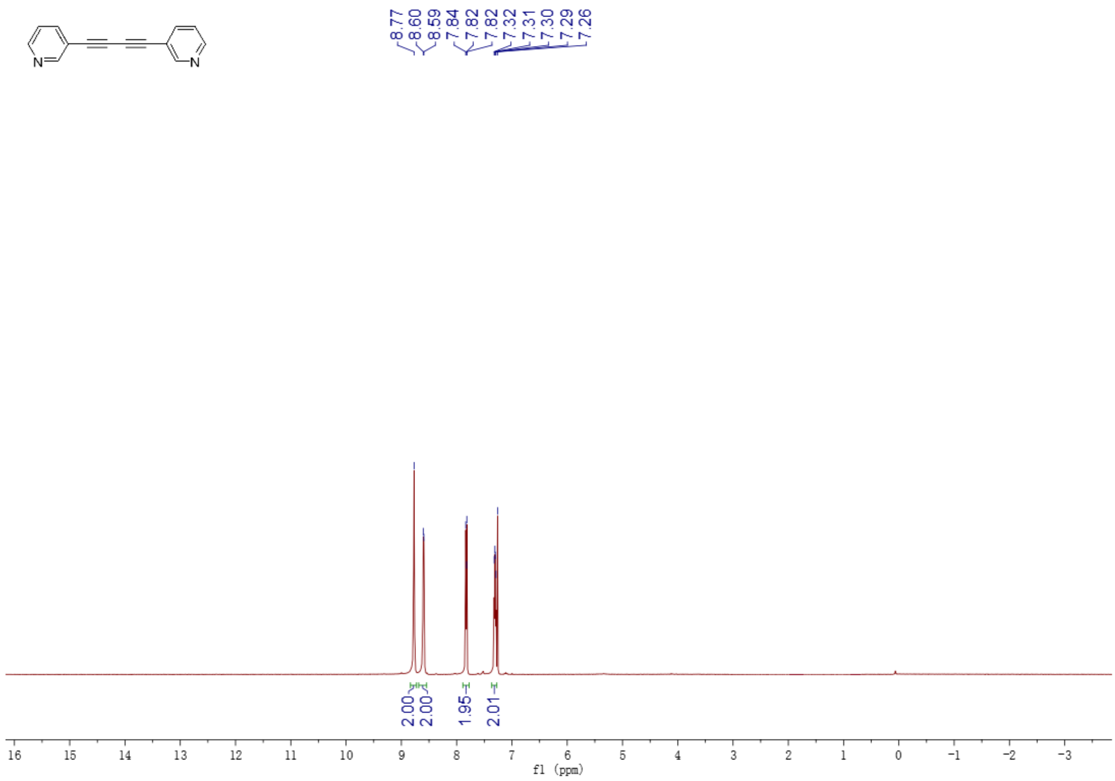

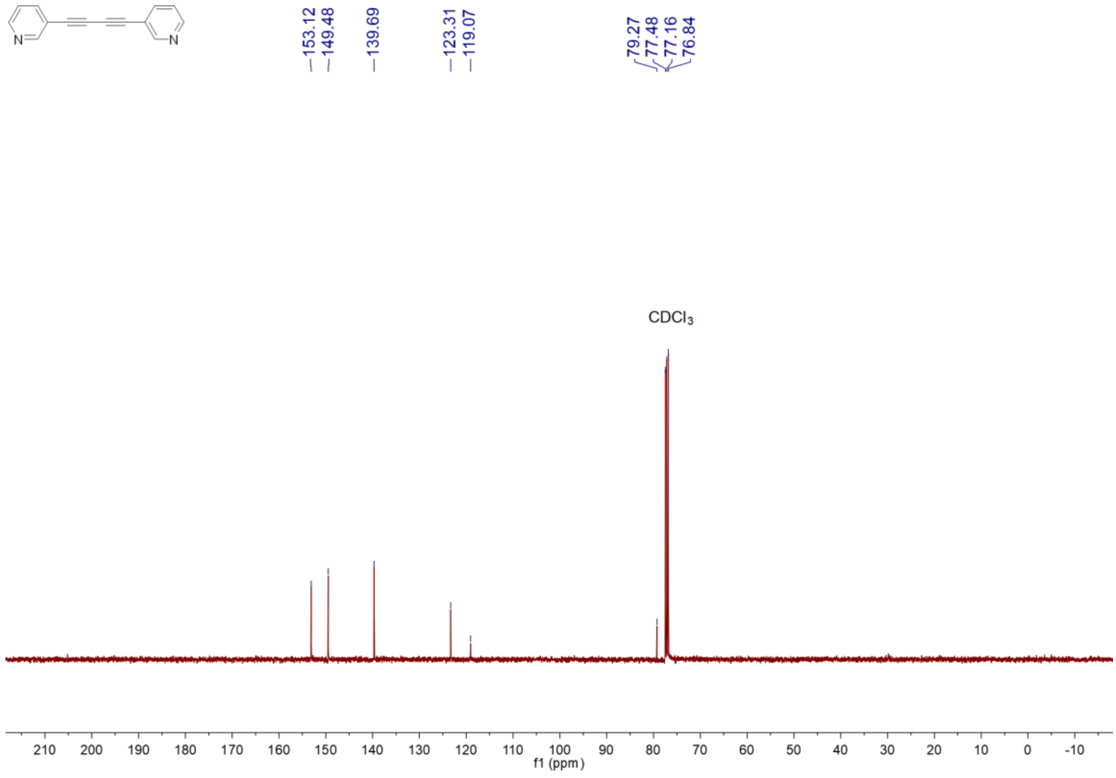

^{1}H NMR (400 MHz, $CDCl_3$) and ^{13}C NMR (100 MHz, $CDCl_3$) spectra of product **4b**

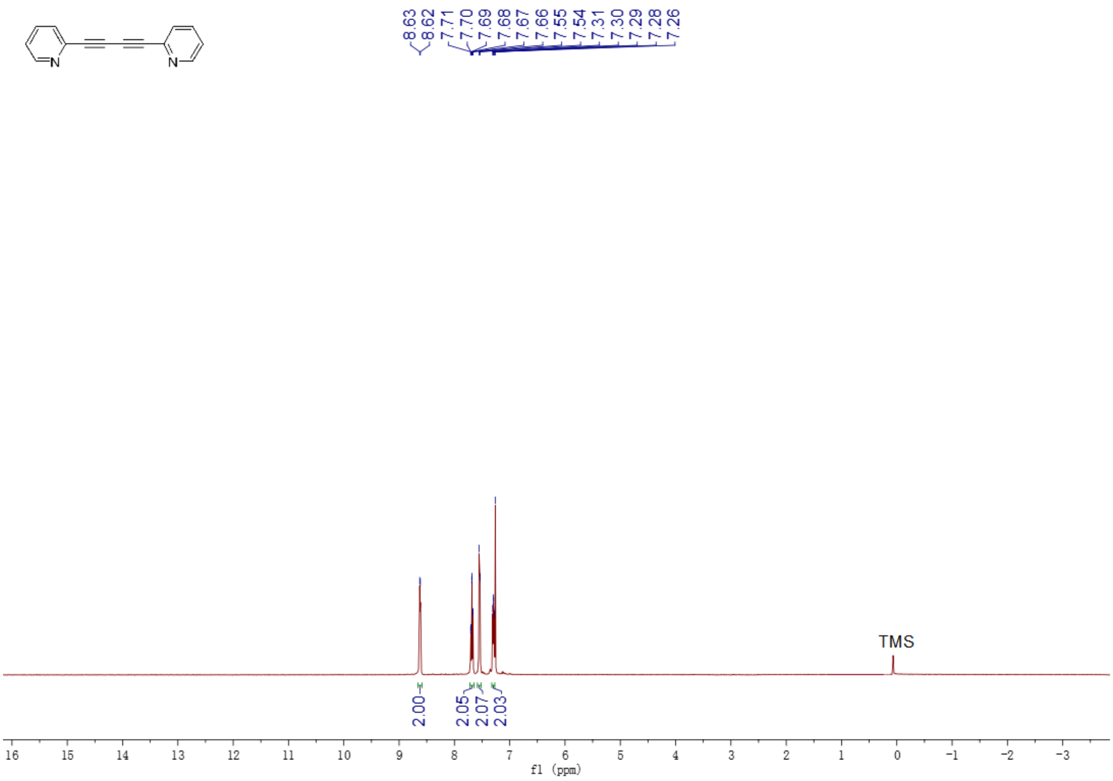

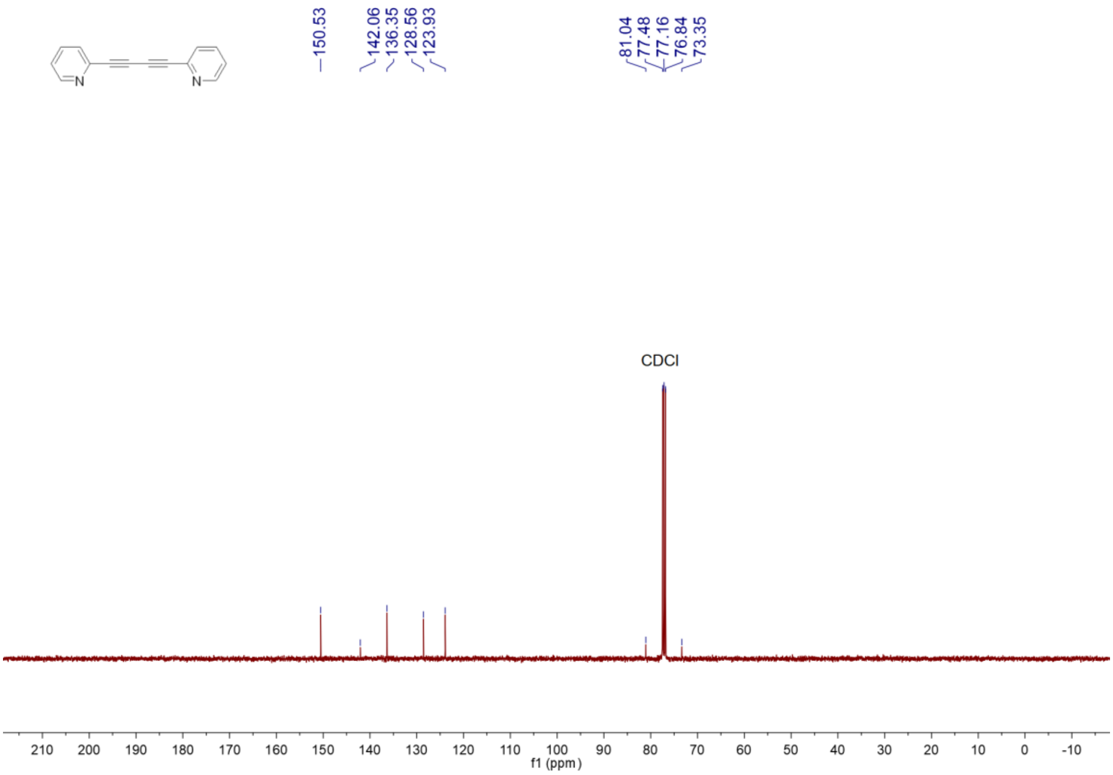

^{1}H NMR (400 MHz, $CDCl_3$) and ^{13}C NMR (100 MHz, $CDCl_3$) spectra of product 4c

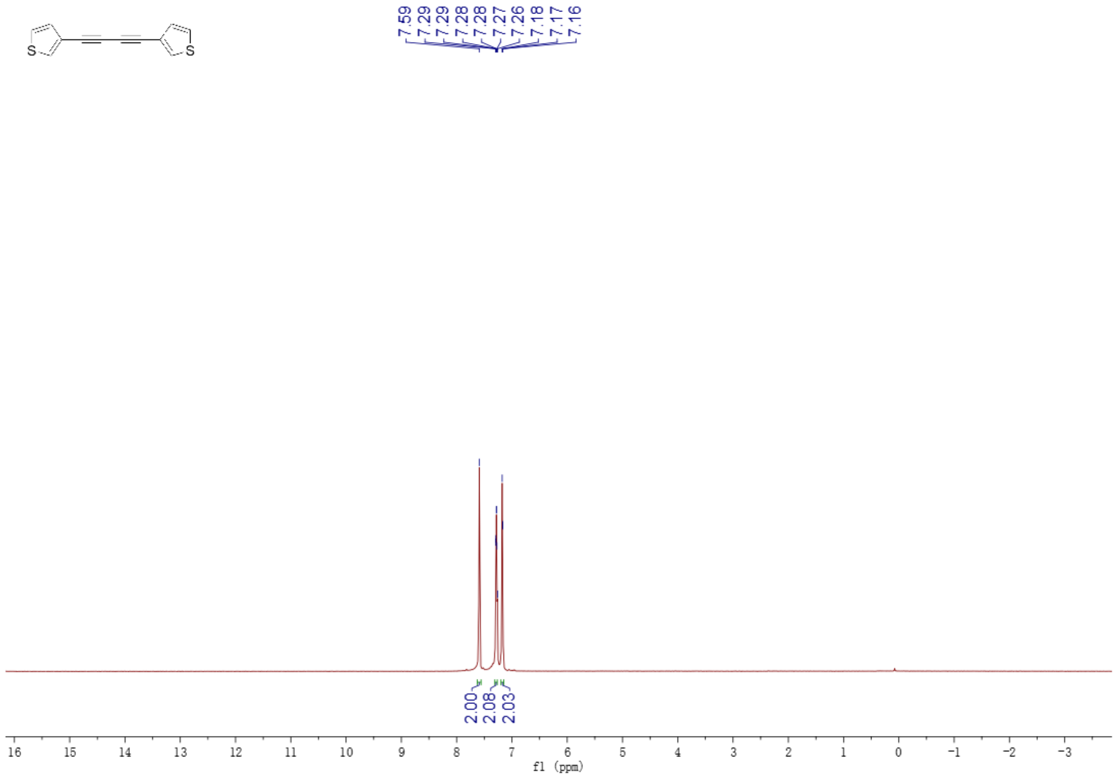

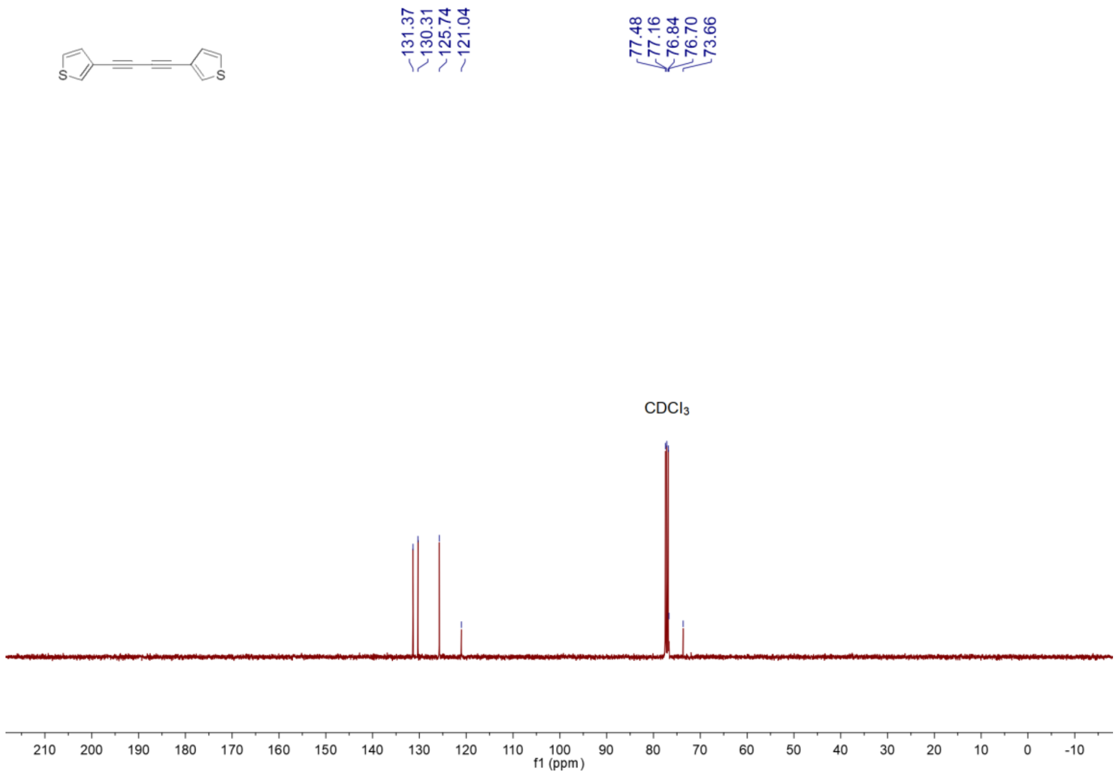

^{1}H NMR (400 MHz, $CDCl_3$) and ^{13}C NMR (100 MHz, $CDCl_3$) spectra of product 4d

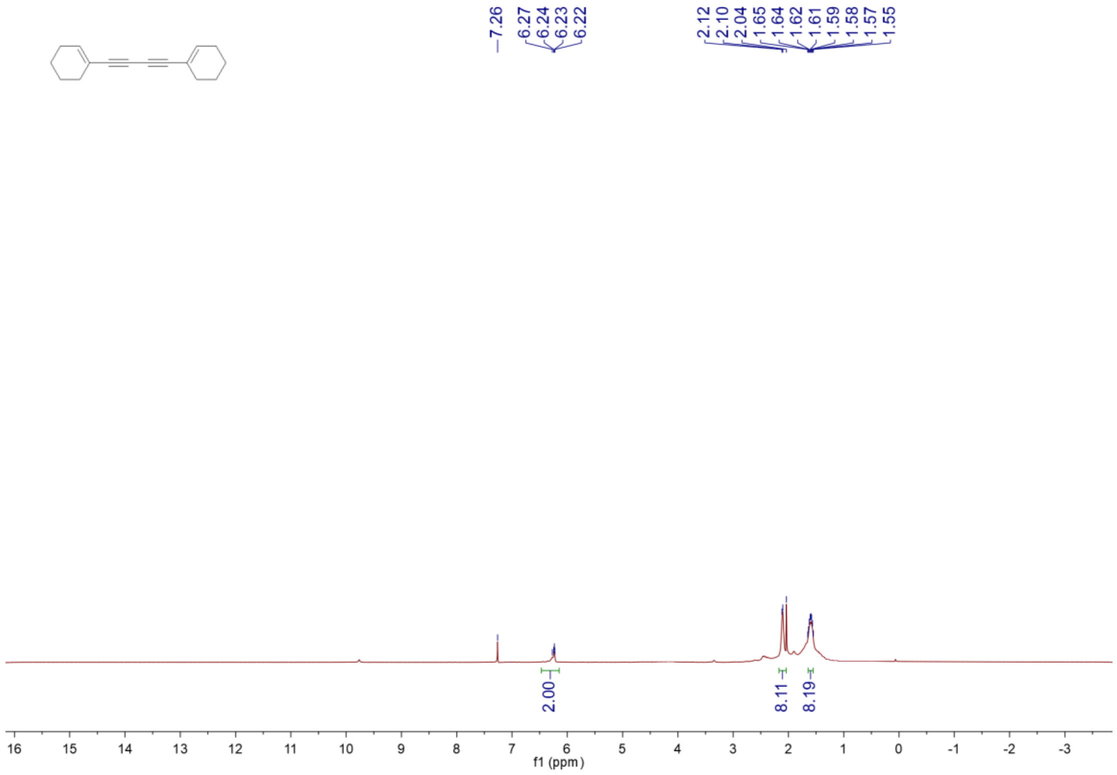

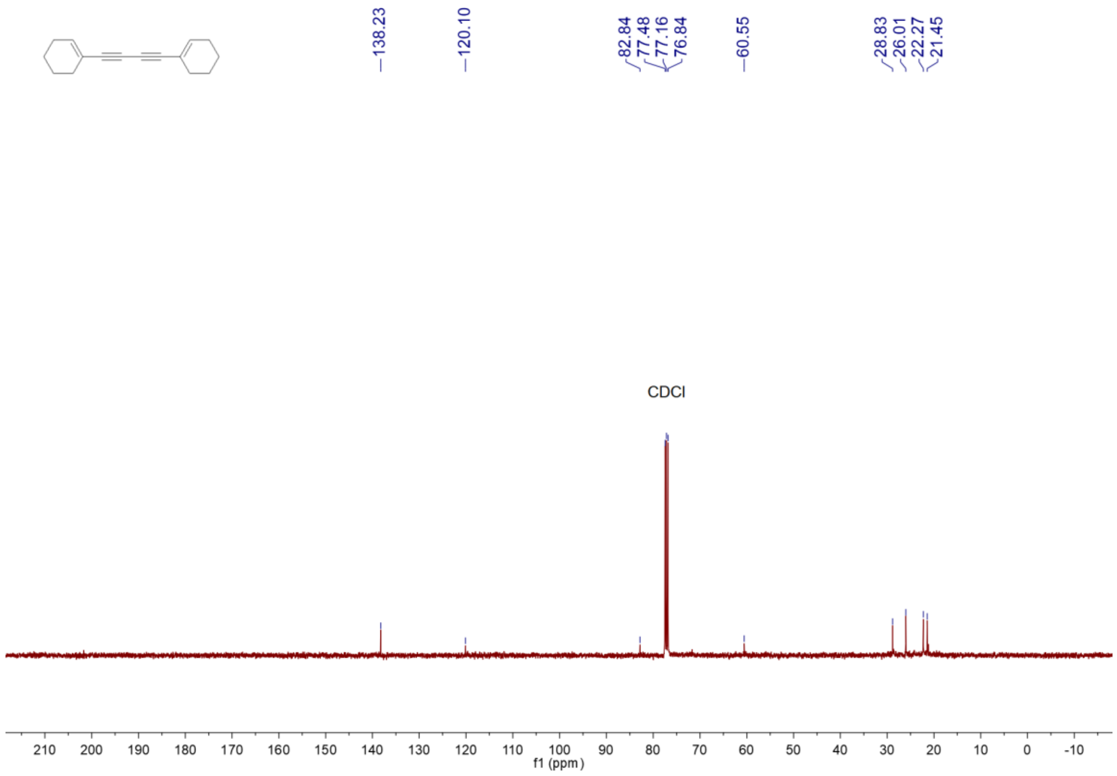

^{1}H NMR (400 MHz, CDCl$_3$) and ^{13}C NMR (100 MHz, CDCl$_3$) spectra of product 4e

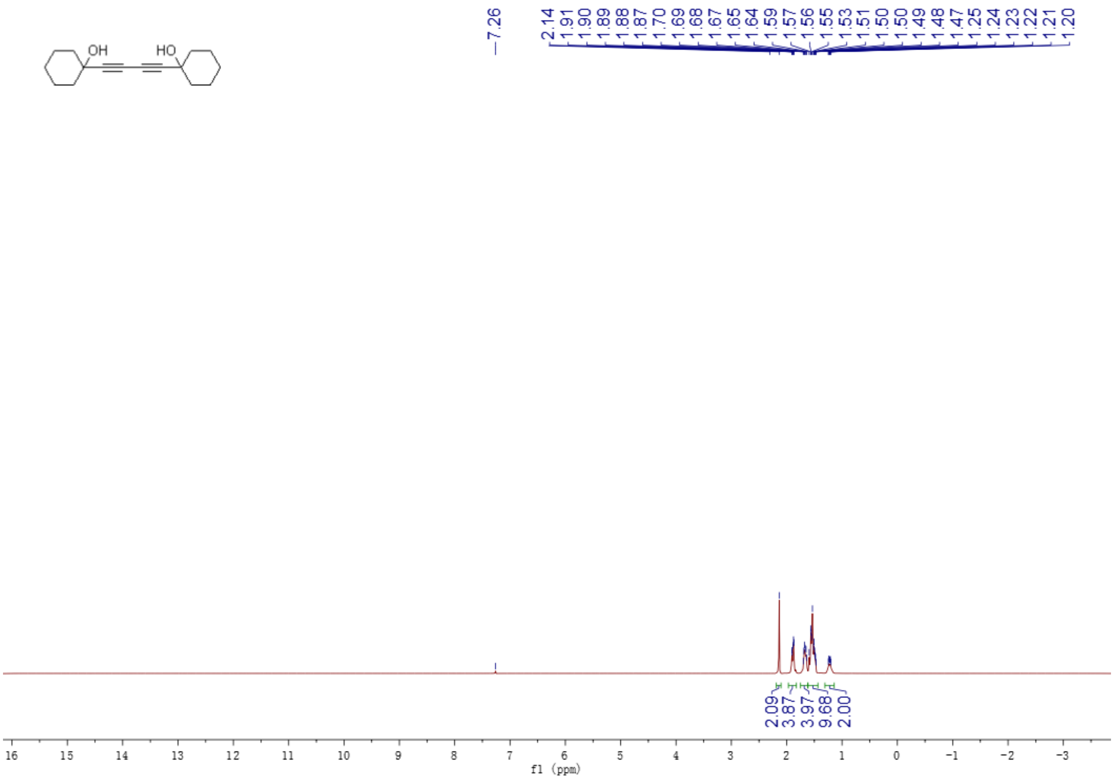

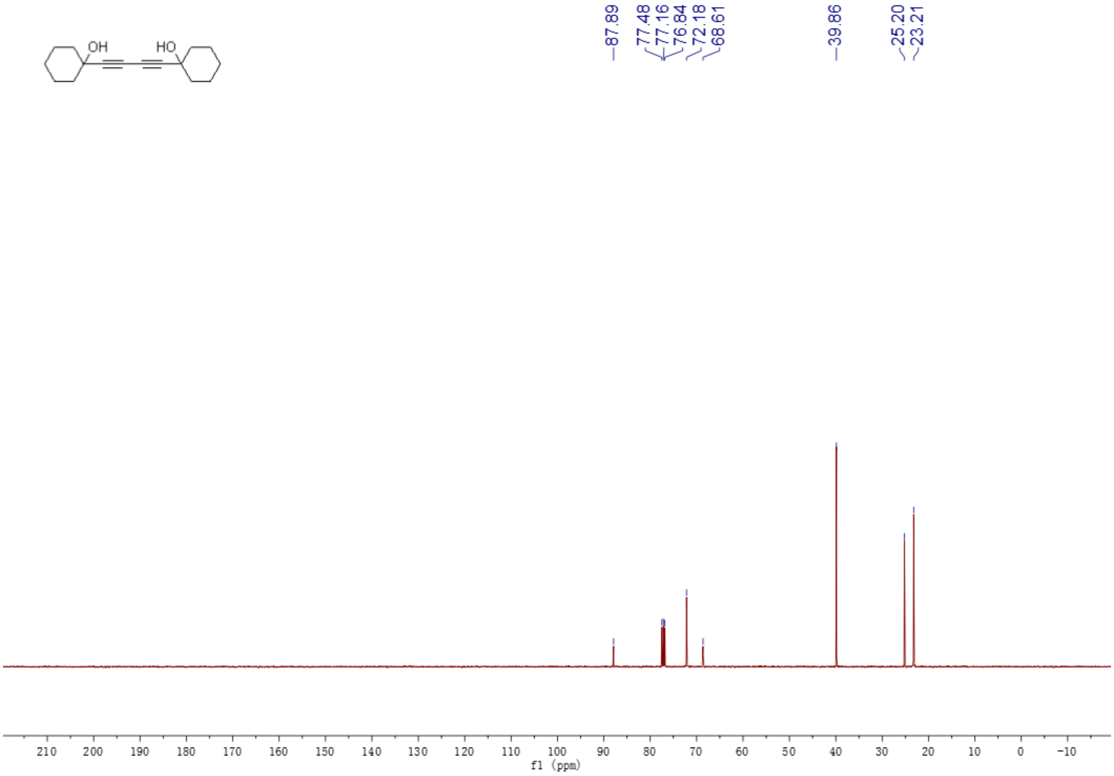

^{1}H NMR (400 MHz, $CDCl_3$) and ^{13}C NMR (100 MHz, $CDCl_3$) spectra of product 4f

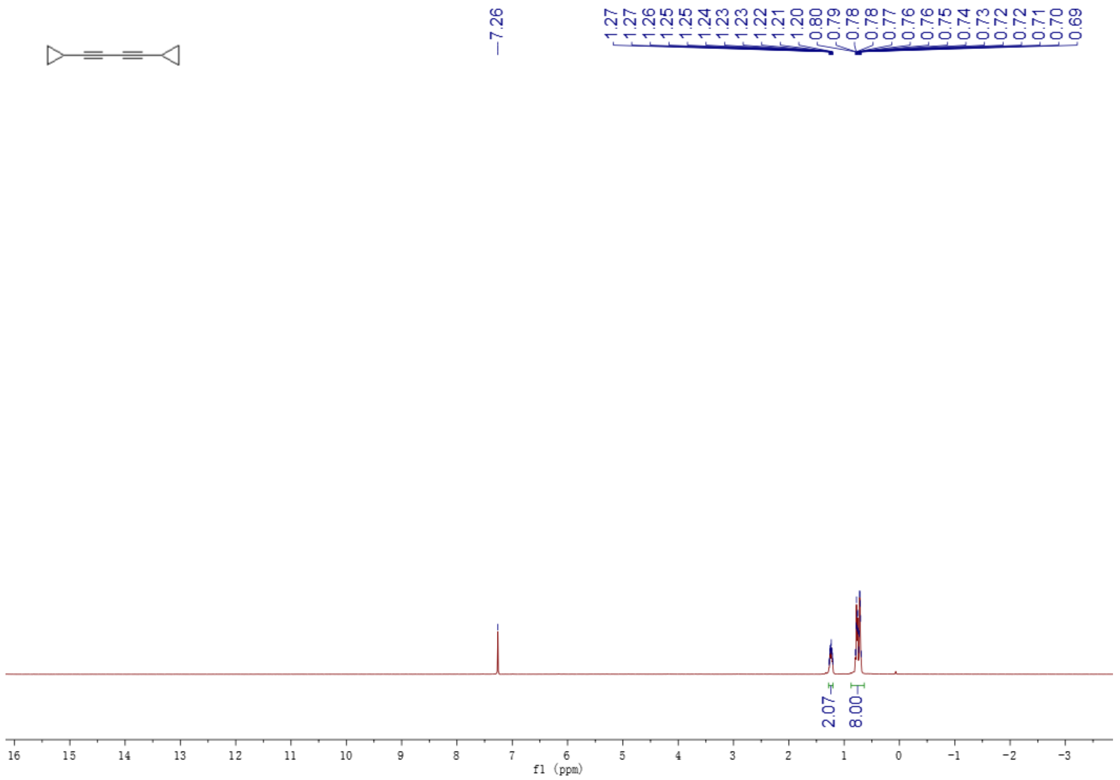

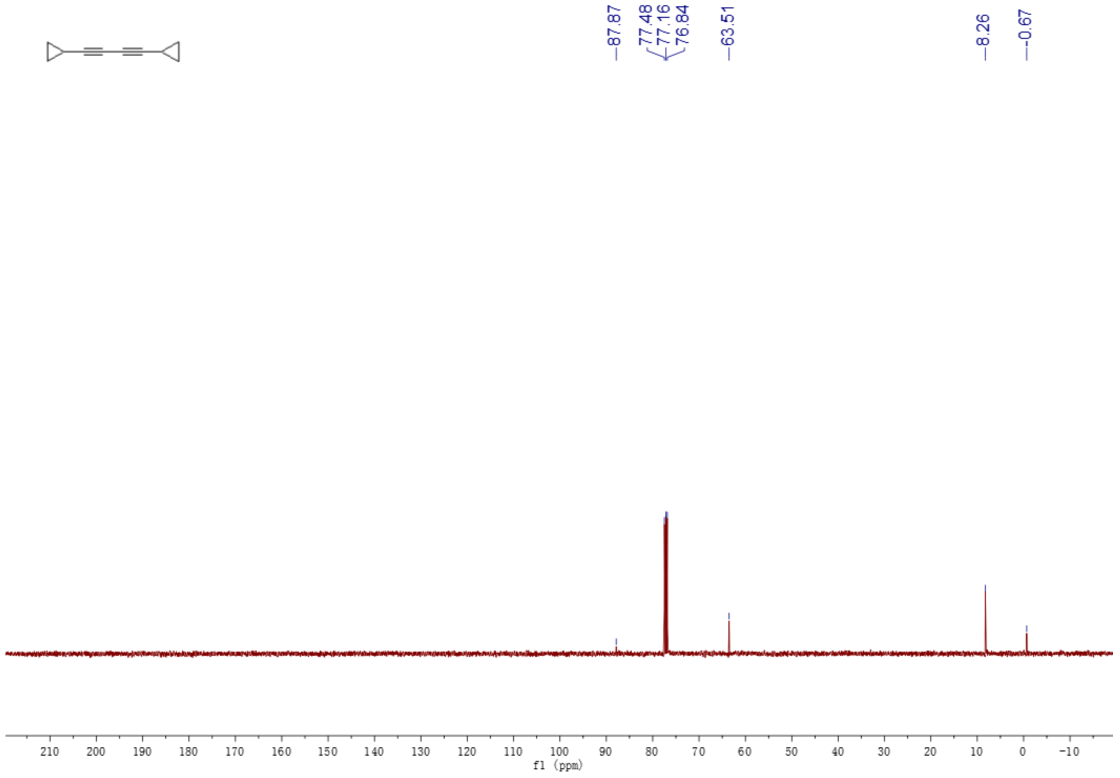

^{1}H NMR (400 MHz, $CDCl_3$) and ^{13}C NMR (100 MHz, $CDCl_3$) spectra of product 4g

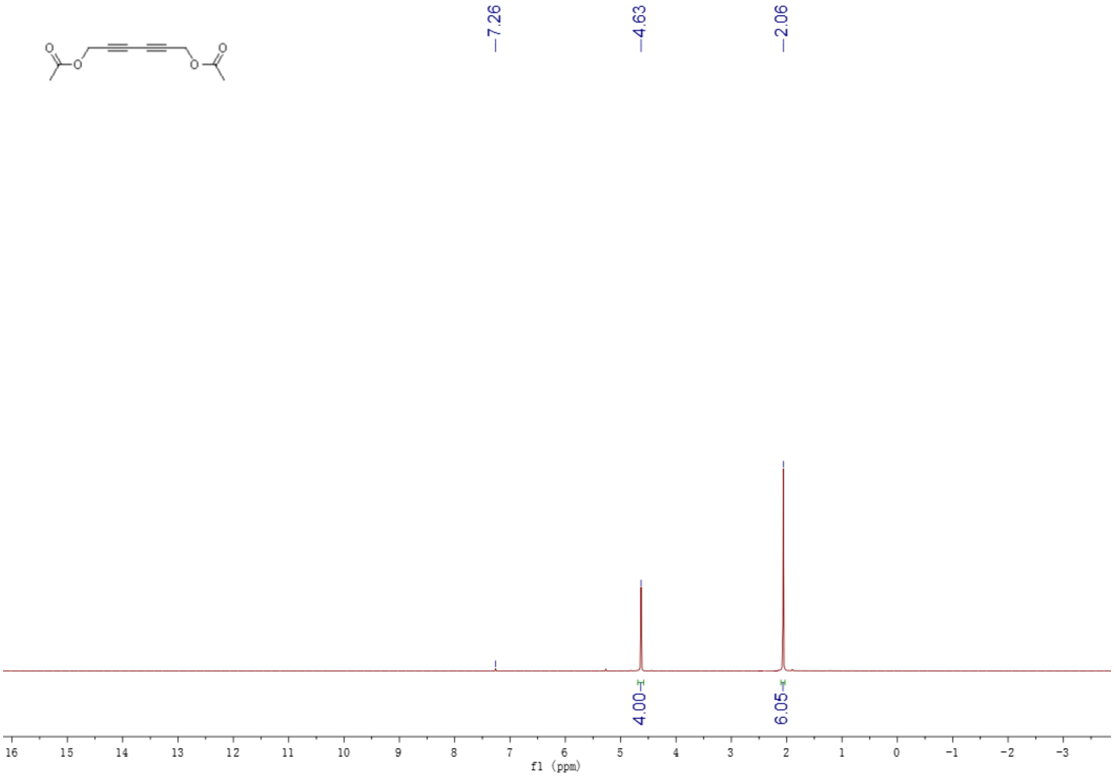

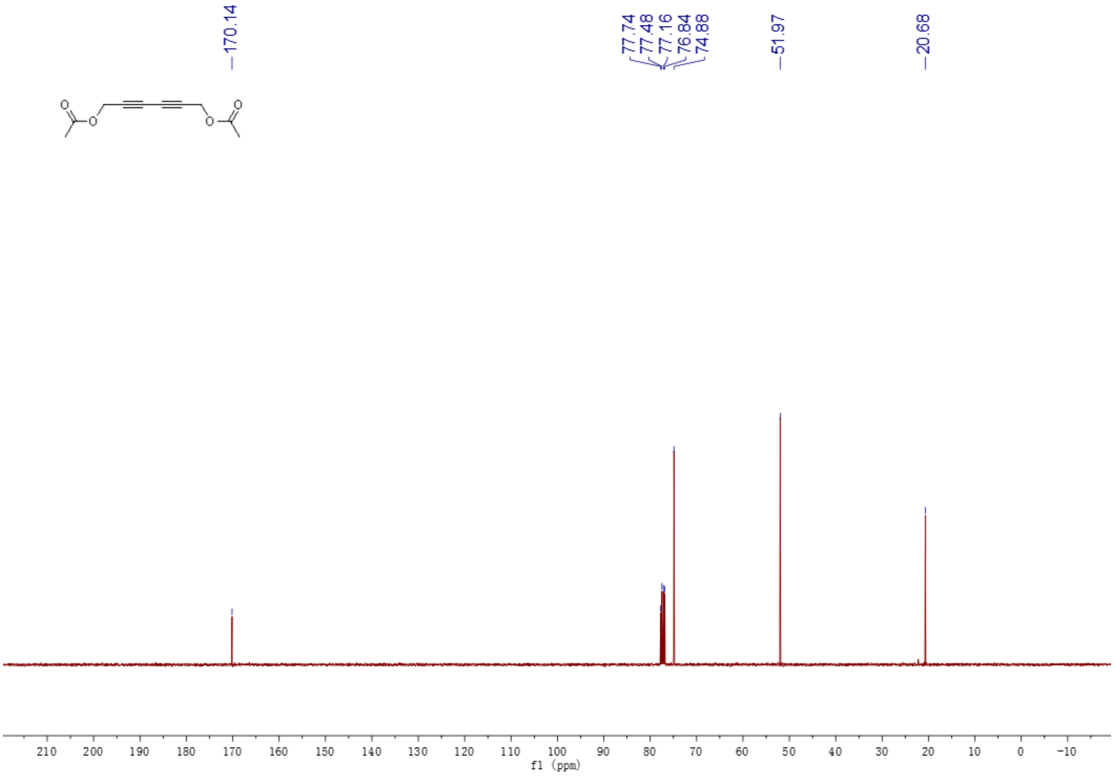

^{1}H NMR (400 MHz, $CDCl_3$) and ^{13}C NMR (100 MHz, $CDCl_3$) spectra of product **4h**

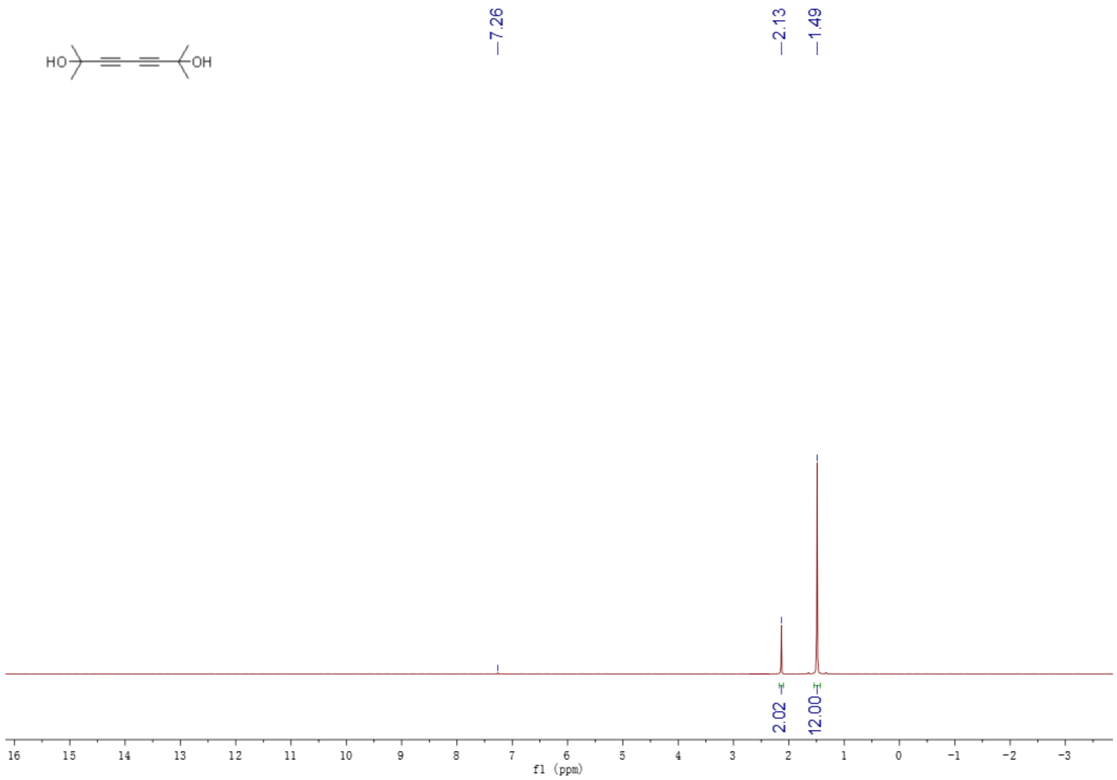

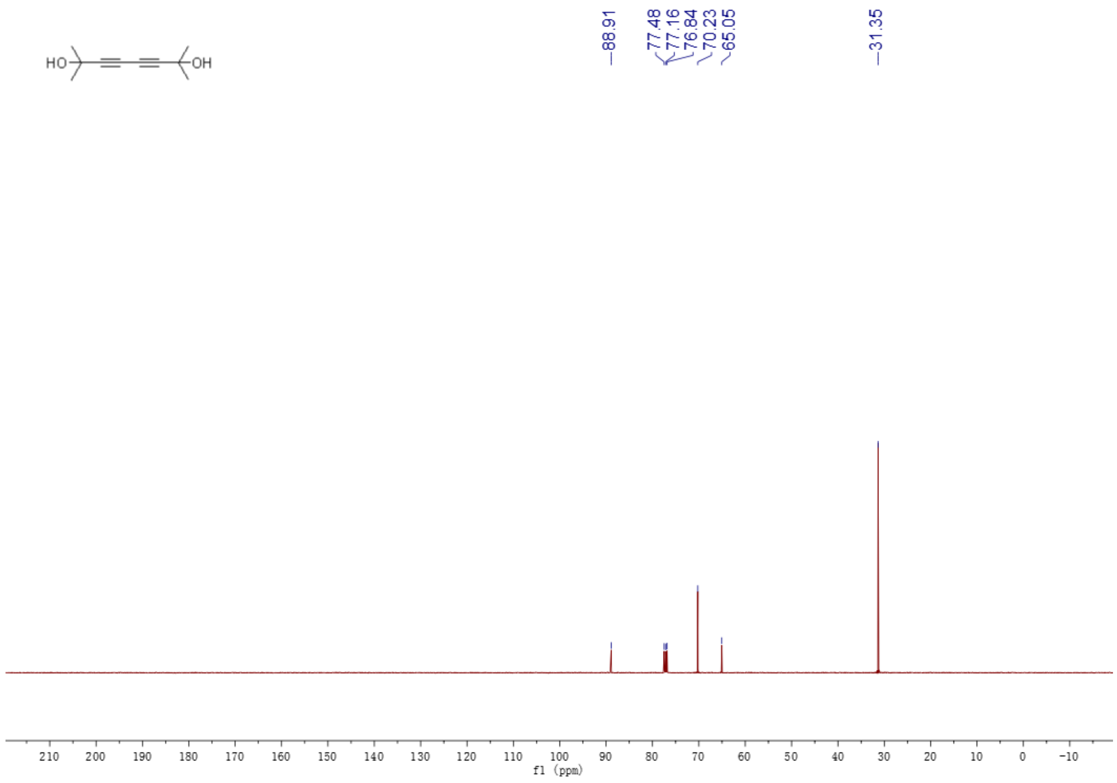

Supplement: Supplementary file 1 [file molecules-28-05083-s001.zip › molecules-2449664-supplementary.pdf]
